# Sustained ash emission during lava effusion is a hidden volcanic hazard of silicic eruptions

**DOI:** 10.1038/s41467-026-76464-w

**Published:** 2026-08-08

**Authors:** Jingwei Zhang, Hugh Tuffen, Fabian B. Wadsworth, Jamie I. Farquharson, Holly E. Unwin, Alastair G. E. Hodgetts, Thomas J. Aubry, Maciej W. Farbicki, Dave McGarvie, Daníel Freyr Sigurbjargarson

**Affiliations:** 1 Lancaster Environment Centre, Lancaster University, Lancaster, UK; 2 Department of Earth and Environmental Sciences, Ludwig-Maximilians-Universität München, Munich, Germany; 3 Institute for Research Administration, Niigata University, Niigata, Japan; 4 British Geological Survey, Nottingham, UK; 5 School of GeoSciences, University of Edinburgh, Edinburgh, UK; 6 Department of Earth Sciences, University of Oxford, Oxford, UK; 7 Nature Conservation Agency of Iceland, Hvolsvöllur, Iceland

**Keywords:** Volcanology, Natural hazards

## Abstract

Volcanic ash poses diverse and widespread hazards. Hybrid silicic volcanism – concurrent lava effusion and explosive ash venting – may constitute a significant but poorly quantified source of ash, so far only documented from a small number of eruptions, most notably the 2011–2012 eruption of Cordón Caulle (Chile). From that eruption, veneers of ash found welded on lava fractures have been attributed to the hybrid activity. Here, we report similar ash veneers from Holocene rhyolitic lavas at Torfajökull, Iceland, and Lipari, Italy. Focusing on the 877 CE Hrafntinnuhraun eruption of Torfajökull, we highlight that these veneers are widespread across its lavas, indicating that, similar to Cordón Caulle, Hrafntinnuhraun experienced long-lived ash venting alongside lava effusion. We develop a quantitative ash capture model based on textural complexities within the veneers, anda material and textural complexitiesrgue that hybrid activity is common to silicic volcanism and therefore represents a substantial and under-recognized hazard.

## Abstract (web summary)

The study presents field evidence that Iceland eruptions of silicic lava are typically accompanied by long-lasting ash plumes but remain hidden in the tephra record. This potential hazard should be appraised for all for silicic lava eruptions.

## Introduction

Silicic eruptions typically begin with an explosive phase^1^. Direct observations at Volcán Chaitén (VEI ∼ 4, 2008, Chile) and Cordón Caulle (VEI ∼ 5, 2011–12, Chile) have shown that following an initial, purely-explosive phase, lava can effuse from the vent while an explosive component of the eruption remains active, in the form of low- to moderate intensity ash venting^2–9^. Similar behavior can also manifest in lower silica magma, including the 2010 eruption of Eyjafjallajökull, Iceland, during which weak but sustained 2–4 km high ash plumes accompanied lava effusion^10^. At Cordón Caulle, continuous ash venting^3^ over ∼300 days sustained a continuous low plume that accompanied the entire duration of lava effusion^11–13^. Videography captured during the lava effusion phase showed that the ash feeding the explosive plume was emitted through meter-scale, arcuate fractures in the lava ’pad’ directly atop the vent^3,8,9,14^. After the Cordón Caulle eruption ceased, the emplaced lava was examined in detail. Open fractures in the lava are commonly coated by reddened, grainy veneers up to a few millimeters thick. These veneers were found to be formed of oxidized, very fine (∼1 µm) juvenile ash, varying from incipiently to thoroughly sintered onto the lava. As such, these veneers were recognized as the unique product of the through-lava ash venting phenomenon^8,9,15^.

Pyroclast-filled fractures (tuffisite) have previously been documented in and around dissected silicic conduits, lavas, and ejected bombs^7,16–21^, with clastic fill deriving from deeper fragmentation^20^ and local wall abrasion^17^. Most tuffisite networks are not directly connected to the surface^21^ and thus provided no pathway for discharge into the atmosphere. However, the fractures observed at Cordón Caulle represent the uppermost termini of tuffisite networks that have reached the surface, and thus do represent an atmospheric venting pathway. Particles are fed from sustained primary fragmentation in the shallow conduit during active lava effusion^8,9,15^. Veneered fractures therefore provide unambiguous evidence of plume-generating, through-lava ash venting during past lava-producing eruptions^8,9^. This veneered texture has so far only been reported in detail from one non-Chilean volcanic system, at Mono-Inyo, USA, although there is preliminary description of similar features in other Cascades volcanic systems^22^. More examples and evidence are needed to ascertain whether ash venting is a commonplace or even ubiquitous phenomenon that magnifies the hazards posed by lava-producing silicic eruptions.

Here, we examine the Torfajökull volcanic complex, Iceland, focusing in particular on the 877 CE^23^ Hrafntinnuhraun (HRN) eruption—a moderately-sized silicic (rhyolitic) fissure eruption, with well-exposed associated lavas^24,25^. This event is the second most recent eruption of Torfajökull, and also its largest in historical times^26^. As such, HRN is paramount for assessing hazards posed by future rhyolitic eruptions within the Torfajökull complex^26^. We report an extensive suite of lava veneers from HRN, sampled across the multiple lavas, from vent areas through to flow-fronts. Detailed characterization of the range of particle sizes captured by the veneers versus those that escape into cogenetic plumes provides new insights into how captured ash relate to hazardous ash discharge into the atmosphere. Furthermore, we report lava veneers from four other Holocene silicic lavas of Torfajökull, as well as at two additional Holocene silicic lavas of Lipari, Italy, which point towards the ubiquity of such veneers. Hence, we propose that ash venting is a common and previously overlooked and/or unrecognized hazardous phenomenon in silicic eruptions. Better understanding of this process can enhance knowledge of conduit processes and improve management of the associated volcanic hazards.

## Results

### Geological background and field observations

**Fig. 1:**
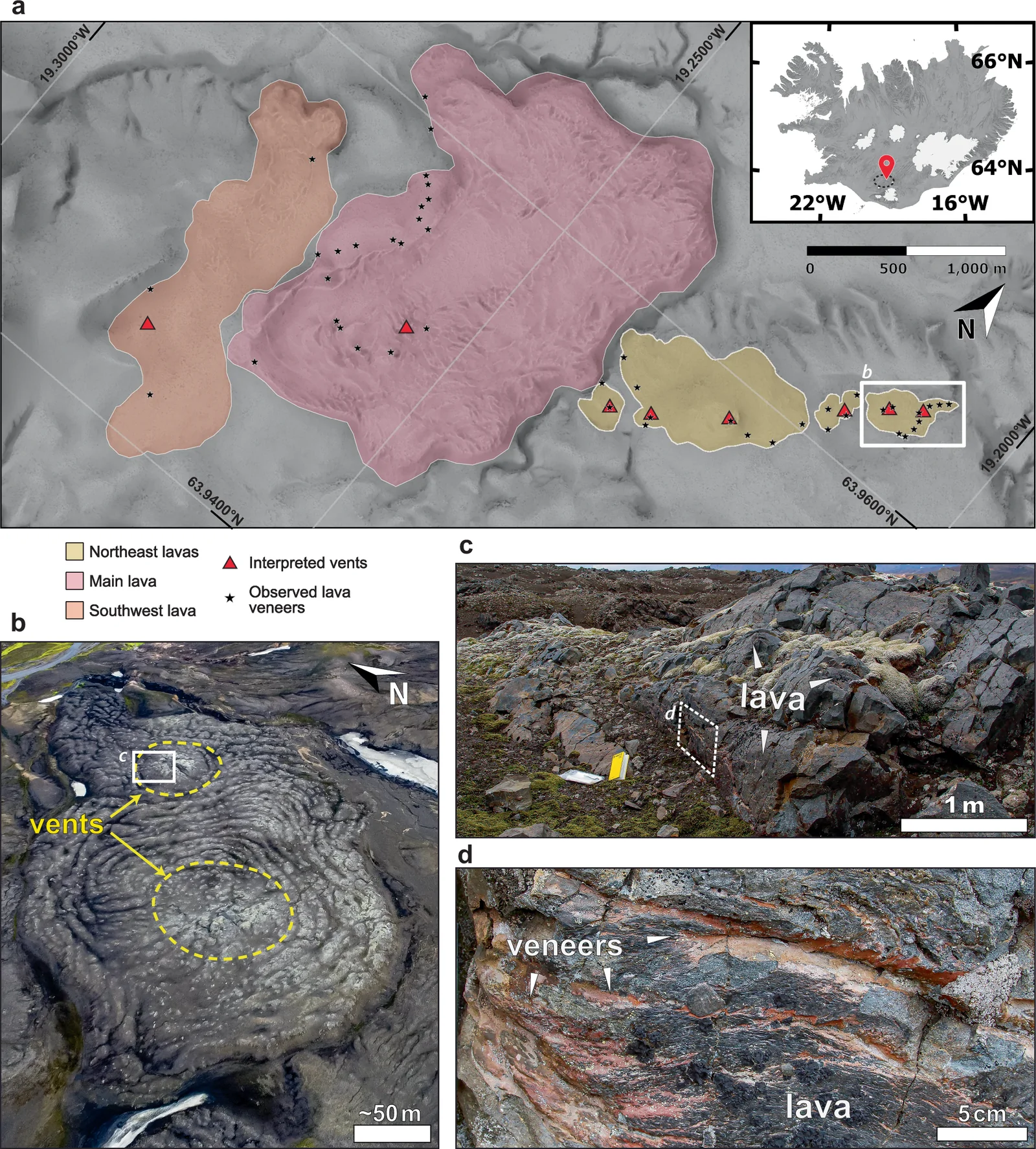
Geological context and field localities of Hrafntinnuhraun. **a** The mapped extent of the Hrafntinnuhraun lavas, interpreted vent locations, and locations of lava veneers as observed in the field, overlaid on a digital elevation model (ÍslandsDEM útgáfa 1.0 by Náttúrufræðistofnun, licensed under CC BY 4.0; accessed via https://gatt.lmi.is); the white rectangle indicates the area shown in (**b**); *Inset*: the location of Hrafntinnuhraun (red pin), within the Torfajökull volcanic complex (dashed outline), Iceland. **b** An oblique-angle aerial photo of part of the Hrafntinnuhraun northeast lavas; white rectangle indicates the area shown in (**c**). **c** A representative outcrop of Hrafntinnuhraun lava with veneers indicated by white arrows; the white dashed outline indicates the area shown in (**d**). **d** A close-up photo of part of the ash veneer outcrop in (**c**), showing a number of streaky, red-colored veneers across the lava fracture surface.

HRN was a 877 CE, VEI ∼3 rhyolite eruption within the Torfajökull volcanic complex^24,26^. Multiple lavas (total volume ∼0.18 km^3^) were fed from a series of localized vents, on an approximately northeast-southwest trending, ∼4 km long fissure^27^ (Fig. 1a). The HRN lavas can be roughly grouped into three areas: northeast (NE lavas), main (main lava), and southwest (SW lava); these are shown on Fig. 1a. The NE lavas comprise a series of adjacent, apparently thin (<15 m), blocky lavas, up to hundreds of meters long. Elevated dome-like protrusions up to ∼50 m wide, roughly co-linear with the fissure, represent vent structures. The main lava is thick (maximum ∼45 m), ∼2 km long, and more topographically complex. The vent(s) for the main lava is likely close to the point of highest elevation on the lava surface, although its precise location or number of vents is unknown. The SW lava is apparently thin (<25 m) and inundated by overlying tephra; similar to the main lava, its vents are presently obscured (Fig. 1a).

Fracture-coating lava veneers were identified across all lavas at HRN, from their vent areas to flow-fronts. For clarity, here we distinguish between tuffisites and veneers, with the former restricted to completely filled veins, and the latter as exposed, coated surfaces. Both are common across HRN lavas and often associated at sample- and outcrop scale.

HRN lavas are predominantly poorly to moderately vesicular and crystal-poor. Both large-scale vent-defining lava fractures and centimeter-scale prismatic joints frequently expose centimeter- to meter-scale patches and/or streaks of millimeter-thick veneers (Fig. 1d). Thin (mm- to cm-scale) internal tuffisites, connected to veneers, are frequently exposed in autobrecciated clasts that are common atop the lavas. Within the (interpreted) vent areas, loosely packed veneered clasts of both lava fragments and pumiceous pyroclasts are common, consistent with a final stage of ash venting through an immobile, poorly consolidated breccia directly above the conduit.

In addition, we surveyed the Holocene Torfajökull silicic lavas Hrafntinnusker (c. 9 ka), Dómadalshraun (c. 150 CE), Laugahraun and Námshraun (both c. 1477 CE)^24,26^, identifying multiple veneers within each. We also visited the Holocene silicic lavas Forgia Vecchia and Rocche Rosse^28^, on the island of Lipari, Italy, identifying lava veneers within both lavas (see below).

### Veneer characteristics and interpretation

**Fig. 2:**
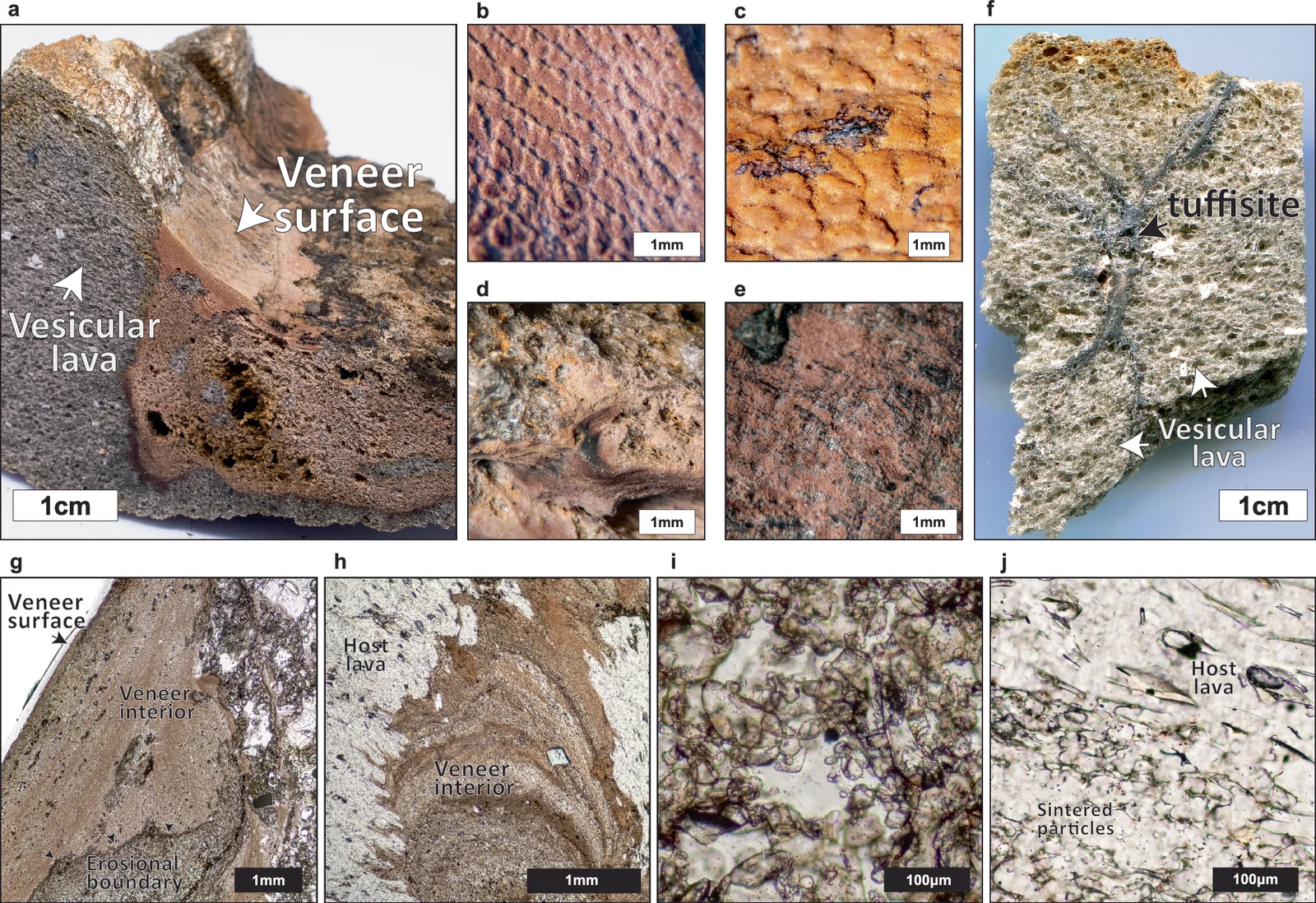
Details of ash veneers on Hrafntinnuhraun (HRN) lava samples. **a** A representative sample of a veneered HRN lava, with cut section orthogonal to the veneer surface. **b** A veneer with millimetric ripple- or dune-like features. **c** A similar feature as shown in b showing apparent sedimentary features on veneer surfaces. **d** A thin veneer overlaying a chaotic fracture surface. **e** A very thin, streaky, veneer. **f** A millimeter-wide, tuffisite within lava, located approximately a few centimeters from a veneered surface. **g-j** Photomicrographs of thin sections of HRN veneer samples under plane polarized light. **g** Veneer material with weakly-laminated interior and a prominent, internal erosional boundary. **h** Veneer material with strongly-laminated interior with a wispy, flame-like contact with the host lava. **i** Weakly-sintered glassy particles in the veneer material. **j** Thoroughly sintered glassy particles in the veneer-connected tuffisite.

More than 50 samples containing ash veneers and/or tuffisites were collected at HRN. Samples were collected from the exposed lava surfaces (uppermost ∼5 m) and the lava flow-top. Hand specimens of HRN veneer samples display wide textural diversity (Fig. 2). Veneer material often thickens into local depressions and, in vesicular hosts, partially infill the vesicle network up to centimeters from the in situ veneer surface (Fig. 2a). Thicker veneers are typically smooth (Fig. 2a) or form dune- or ripple-like structures (Fig. 2b, c) while thin veneers more closely trace the fracture surface, forming uneven and/or streaky patterns (Fig. 2d, e). Hardness of the veneer material varies greatly between samples—from readily scratched by fingernail, to similarly-indurated to dense lava. Veneer surfaces rarely display evidence of late-stage brittle fracture, suggesting few (if any) are fractured internal tuffisites.

Transmitted light microscopy reveals that veneer interiors are texturally complex (Fig. 2g, h). Veneer material comprises predominantly glassy, rounded particles that display moderate sintering, with rare crystal fragments (Fig. 2i). In sections perpendicular to the veneer surface (i.e., into the lava), veneer-hosting lava commonly displays curving, millimeters-thin internal tuffisites, tens of centimeters away from—yet remaining connected to—the veneers’ in situ surfaces (Fig. 2f). When viewed in thin section, these anastomosing features are particulate, albeit sintered to a relatively advanced degree (Fig. 2j).

Energy dispersive X-ray spectroscopy (EDS) analyses were carried out on thin sections of five representative veneered HRN samples to fingerprint the compositions of the veneer material (Supplementary Table 1, Fig. 2). Major element compositions of veneer particles and corresponding lava hosts are virtually identical, as well as consistent with literature values for Torfajökull rhyolites^25,27^. This is consistent with the veneers comprising juvenile ash particles co-genetic with the host lava^9^, as opposed to a secondary origin (e.g., vapor-phase mineralization).

**Fig. 3:**
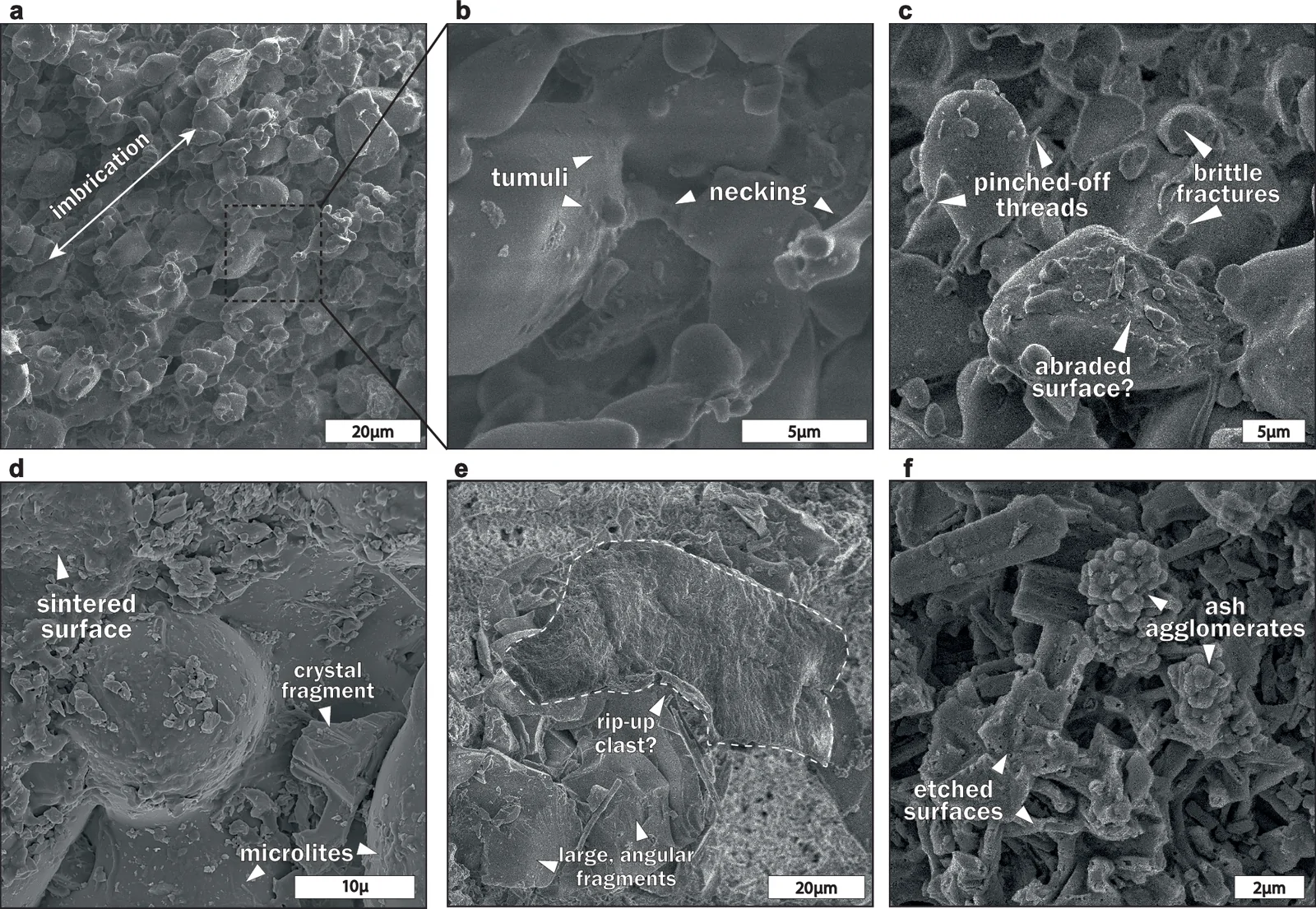
SEM images of Hrafntinnuhraun veneer surfaces, showing the textural diversity of ash veneers. **a** A surface of an HRN lava veneer, showing moderate alignment of particle elongation which we interpret as imbrication fabric; dashed box indicates field of view of (**b**). **b** Tumuli and necking between particles. **c** Evidence of re-entrainment (pinched-off threads) and mechanical abrasion. Note subsequent particles adhered to the abraded surface. **d** Interior of a tuffisite pocket within HRN lava, showing a range in extent of sintering, including a strongly sintered surface. **e** Surface of a veneered HRN in-vent breccia clast, with several large, angular, brittle-fragmented glassy particles. Note also the apparently ripped-up veneer material, itself composed of sintered particles. **f** Surface of a veneered HRN in-vent breccia clast, showing particles with etched surfaces, with multiple ash agglomerates. Note abundance of tabular—instead of rounded—particles, which are probably crystals.

Using scanning electron microscopy, we show that the veneers comprise µm-sized, glassy juvenile particles at varying stages of sintering (Fig. 3)—from incipient formation of necks (poorly sintered), through to undulatory, smooth surfaces with relict signs of particulate origin (thoroughly sintered), consistent with observations from Cordón Caulle^9^. Particles in several samples display aligned elongation, often with taut, µm-thin threads between particles (Fig. 3a, c), comparable to imbrication fabrics of ignimbrites^29^. Particle morphologies range from prismatic/tabular, to near-spherical, to hemispherical ‘tumuli’ (Fig. 3b), to agglomerates of tens of smaller particles (Fig. 3f). Particles commonly display necking at interfaces (Fig. 3b). Some sintered surfaces display tabular pits, similar in appearance to microlite laths, interpreted as corrosive etching of the microlites from the glass by volcanic gases^9^ (Fig. 3f).

### Particle sizes

**Fig. 4:**
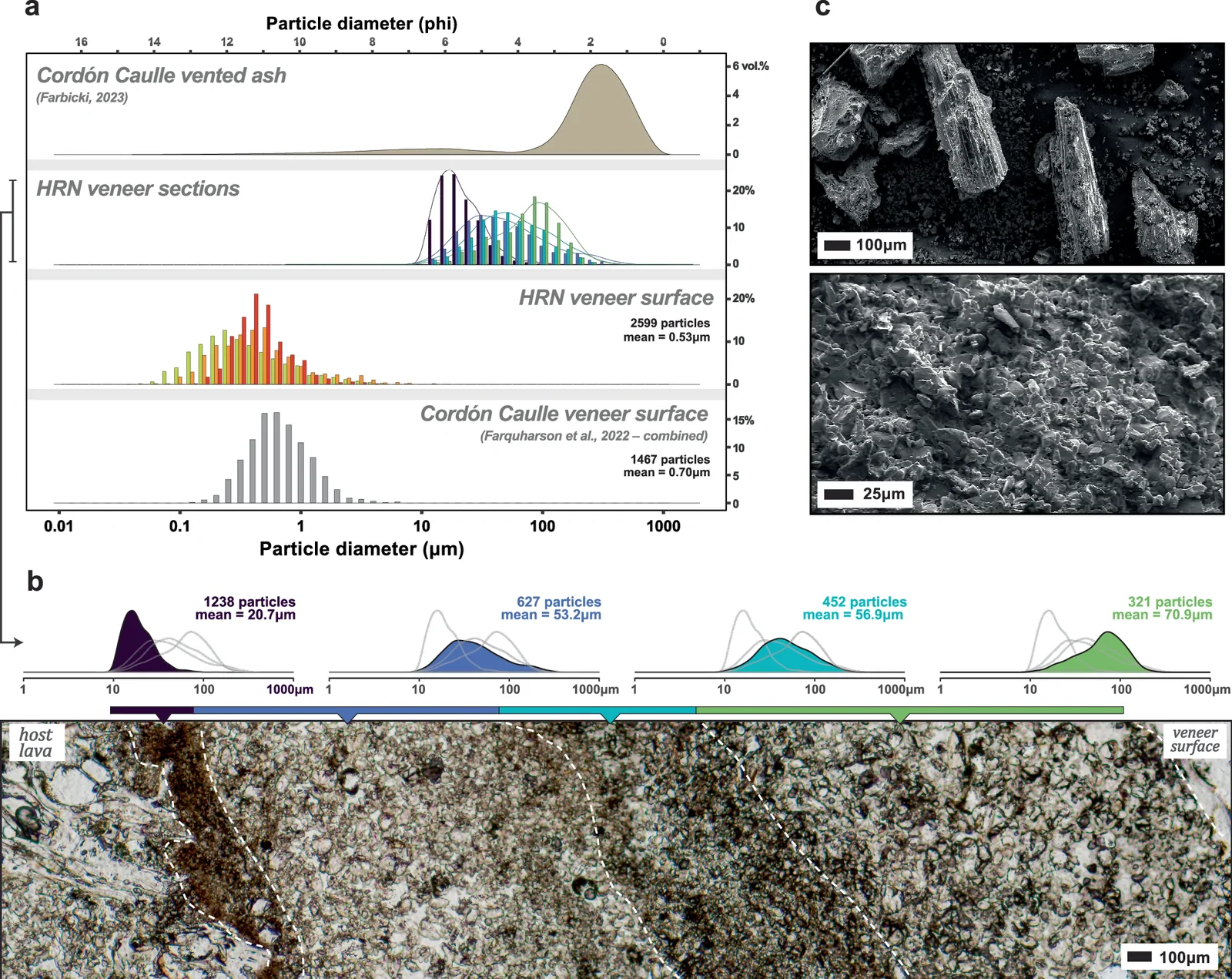
Characteristics of tephra trapped on lava veneer surfaces. **a** Particle size distributions (PSDs) of veneer materials from Hrafntinnuhraun and Cordón Caulle and vented ash from Cordón Caulle. Note that the PSD of vented ash (top) is volumetric, while the others are in count-based histograms. **b** Transmitted light photomicrograph of Hrafntinnuhraun veneer section, annotated with regions within which the PSDs are collected (the relevant PSD is also repeated from a for each part of the photomicrograph). **c** SEM images of Cordón Caulle vented ash (top) and veneer surface (bottom).

Comparison of the particle sizes of captured and vented material can yield important insight into how the shallow pathways modulate the ash populations produced by fragmentation^9^, and is important if the presence of veneers is to be used to infer past ash venting activities. Readily apparent from thin sections and SEM images of the in situ surfaces (e.g., Figs. 2 and 3), HRN veneers host a wide range of particle sizes and size distributions (PSDs), which we analyzed from 2D images (see “Methods” and Supplementary Figs. 3, 4). The PSDs of HRN veneers’ in situ surfaces (mean particle diameter ∼0.5 µm) closely match their counterparts from Cordón Caulle (mean diameter ∼0.7 µm^9^, Fig. 4a). However, HRN veneer interiors comprise a much wider range of particle sizes, from ∼1 µm (i.e., similar to the in situ surfaces), up to ∼100 µm.

As the ash venting products from HRN could not be easily distinguished from the pre-lava explosive phase in the proximal tephra deposits, we instead analyzed the volumetric PSD of a sample of vented ash of Cordón Caulle using laser diffractometry. The PSD of the vented ash encompasses the full ranges of particle sizes observed within the HRN veneers (surface and internal) and further contains a coarser (≳100 µm) fraction that is volumetrically dominant. Indeed, over 80 vol% particles of the vented ash sample were >100 µm in diameter, i.e., larger than those within the veneers (Fig. 4a). We speculate that this is not significantly different from that of the vented material of HRN.

## Discussion

### Particulate flow and fine ash capture during ash venting

The contrasting PSDs of the veneers (surface and interior) and the vented ash present a challenge to attribute these different ash populations to the same source. This is especially pertinent for understanding past eruptions as their venting-phase tephra would rarely, if ever, be preserved and recognizable^30^. A robust capture mechanism is needed to unify these observed PSD ranges, such that we can link the grain sizes of the vented tephra associated with a lava veneer. Turbophoresis—lateral migration of particles from areas of relatively higher turbulence to areas of relatively lower turbulence^31,32^—has been suggested to drive the preferential capture of fine-grained material during ash venting^9^. Specifically, within a flow regime window bounded by Stokes numbers corresponding to theoretical maximum and minimum flow structure lengthscales, discrete ash fractions occupy an optimal parameter regime where both turbophoretic lateral migration and inertial impaction due to decoupling from the carrier phase are operative and effective^9^, likely enhanced by particle disruption (sensu^33^). However, this effect has not been tested in the context of the larger, more volumetrically dominant particles of the veneer interior.

A key unknown in existing models for ash deposition in lavas is the propensity of ash to stick. If we turn to experiments for glass particles sticking (or not) to a surface^34^ we see that the fraction of the total glass particles that stick is temperature dependent, by *f* = *n*_*s*_*/n*_*T*_, where *n*_*s*_ is the number of stuck particles, and *n*_*T*_ is the total number of particles. By using the temperature dependence of viscosity *µ* for the glass particles (i.e., the viscosity of the glass itself, not of the flow containing the particles), we can translate the temperature dependence of sticking to a viscosity dependence of sticking, which is better aligned with generalized sticking theory^35^. We find good agreement between experimental data^34^ for *f* by suggesting *f* = exp(−*µ/µ*_*c*_), where the critical viscosity *µ*_*c*_ = 3.3 × 10^4^ Pa s is a constant we fit for. This has the expected property that *f* → 1 when *µ* < *µ*_*c*_ and that *f* = 0 at *µ* > *µ*_*c*_. This provides a functional method to estimate *µ*_*c*_ below which sticking will occur. Details of our model calibration are given in the Supplementary Information (Supplementary Fig. 4 and descriptions therein).

For our system, we assume that the critical viscosity is related to the kinetic energy of the particle^34,35^ *E*_*k*_, by *µ*_*c*_ = *ξE*_*k*_^*w*^, with *ξ* = 5 × 10^−12^ (in units of kg^1−*w*^ m^−1−2*w*^ s^−1+2*w*^, assuming *w* is dimensionless) and *w* = −1.78 [ref. ^35,36^]. Approximating as spherical particles, we can state *µ*_*c*_ = *ξ*[4*πρr*^3^*u*^2^*/*3]^*w*^, where *ρ* is the density of the particles, *u* is the particle velocity, and *r* is the particle radius. We estimate the viscosity of the glass particles as *µ* = 7.1 × 10^8^ Pa s, using a viscosity model^37^ at a temperature of 900 °C^38^, and magmatic water content of 0.1 wt% (i.e., fully degassed^15^). The initial particles (i.e., prior to sintering) were likely pumiceous (Fig. 4c)—similarly porous compared to the vented ash (up to 67 vol%; [ref. ^3^]), hence *ρ* = 2300 × (1 − 0.67) = 759 kg m^−3^, using 2300 kg m^−3^ as a representative density^3^ for rhyolitic glass.

We find that the very finest particles (≲1 µm), primarily observed on the veneer surfaces, are likely to stick upon impact at even the maximum expected velocity (100 m s^−1^), while larger particles (≳ 10 µm) such as those observed to dominate the veneer interior, should rebound (and likely re-entrain) at flow velocities above ∼5 ms^−1^. The largest particles identified in the veneer material (∼100 µm) will only stick at impact velocities less than ∼0.2 ms^−1^.

At these lower velocities, the flow may no longer be turbulent, and as such turbophoretic effects would wane. We reassess the fluid dynamics within the fracture pathways at these lower flow velocities—where the sticking of volumetrically dominant larger particles *could* take place—by evaluating the Reynolds number (Re), given by *Re* = *u*_*f*_*L/v*, where *u*_*f*_ is the mean velocity of the flow, *L* is the characteristic lengthscale (here, the width of the fracture pathway), and *v* the kinematic viscosity of the fluid (taken to be 1.4 × 10^−4^ m^2^ s^−1^, [ref. ^9^]). Fully developed turbulence is expected at Re > 2900, while laminar flow is expected at Re ≤ 2300 [ref. ^39^]. We note that these thresholds are specific to flow in a pipe, which we use as a reference case for ascent through the shallow surface, but we acknowledge that the exact threshold in terms of Re for fully developed turbulence could be different (e.g., depending on surface roughness or geometry differences such as flow up a crack rather than a pipe). The order of magnitude of these transitions tends to be similar such that turbulence occurs at Re > O(10^3^). Constraining the widths of the fracture pathways during active venting is less straightforward, but generally accepted to be within centimeters to meters^3,9,14^.

**Fig. 5:**
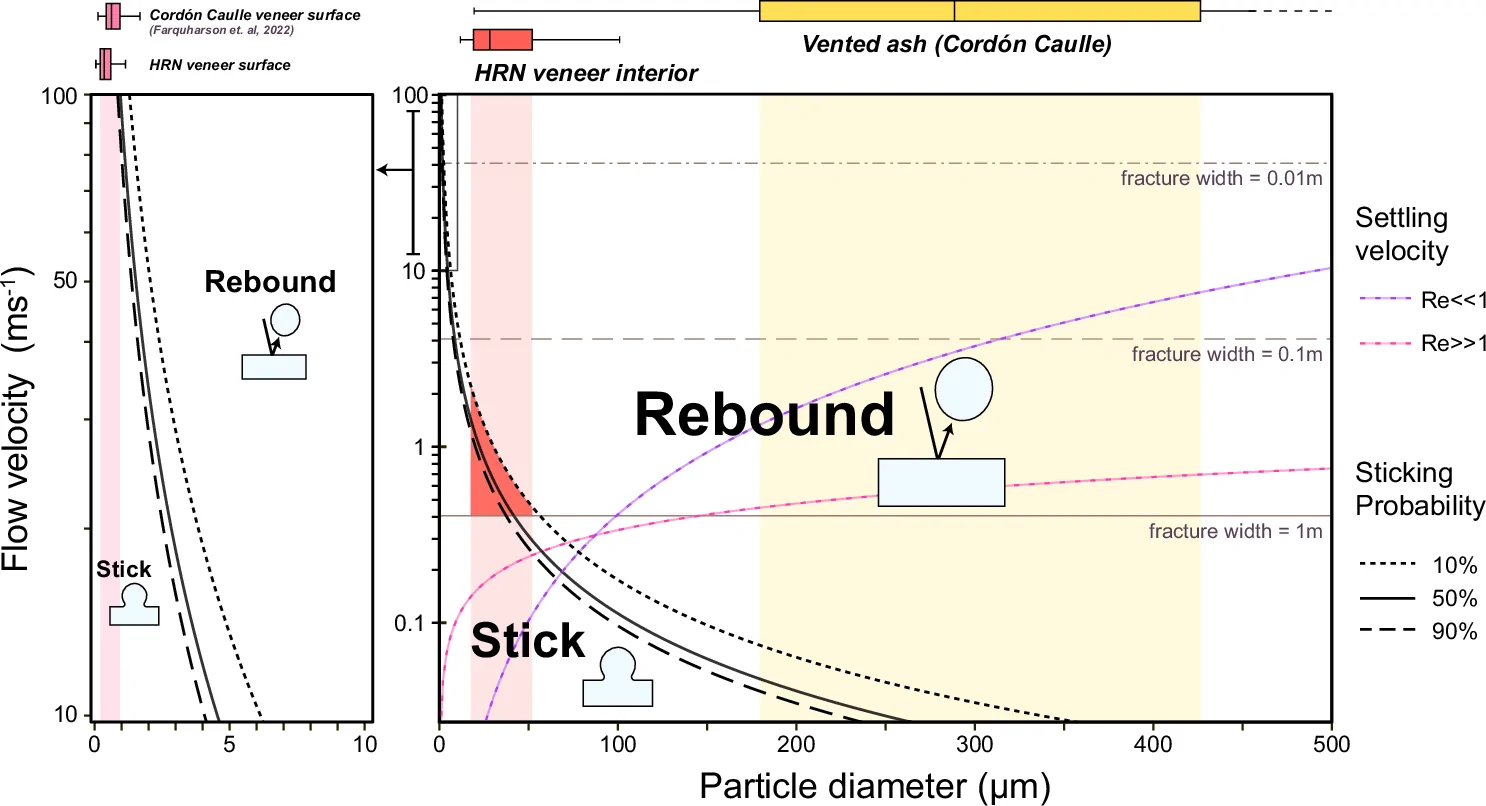
A model for capture of ash particles by turbophoresis-assisted sticking. Two regimes of particle behavior upon impacting the pathway wall are visualized—at lower velocities and smaller particle sizes, the particle sticks upon impact. By contrast, at higher velocities & larger particle sizes, the particle rebounds and is likely re-entrained. The flow turbulence threshold velocities are given by a threshold Reynolds number (Re = 2900) and are plotted for fracture pathways of widths 0.01, 0.1, and 1 m. The three main observed particle size ranges (see Fig. 4) are also shown above the figure as box-and-whisker plots, with the 25th–75th percentile range indicated on the plots. Settling velocities (as approximations for the fluidization velocity) for laminar (Re < 2300) and turbulent (Re > 2900) flow conditions are also plotted. The condition window favoring capture of representative HRN veneer interior particles is highlighted.

We also evaluate the settling velocities *v*_*s*_ of particles to serve as minimum fluidization velocities^40^, assuming spherical particles of *ρ*_*p*_ = 759 kg m^−3^ (see above), and a representative gas viscosity of 1×10^−5^ Pa s. We calculate *v*_*s*_ for laminar flow conditions (Re < 2300) by

1 $${v}_{s}=\frac{2\left({\rho }_{p}-{\rho }_{g}\right)g{r}^{2}}{9{\mu }_{g}}$$

where (in S.I. units) *g* is the gravitational acceleration, *r* is the clast radius, *ρ*_g_ is the gas density, and *µ*_*g*_ is the gas viscosity; for turbulent flow conditions (Re > 2900) by

2 $${v}_{s}=\sqrt{\frac{8\left({\rho }_{p}-{\rho }_{g}\right){gr}}{3{C}_{D}{\rho }_{g}}}$$

where *C*_*D*_ is the drag coefficient (taken to be 0.44 for fully developed turbulence)^41^. We use a gas density of 18.5 kg m^−3^ for both equations, the ideal gas law of *ρ*_g_ = *pM*/(*RT)*, where *p* is the pressure (taken to be 1.0 × 107 Pa), *M* is the molar mass in kg mol^−1^ (taken to be 0.018 kg mol^−1^, i.e., that of water), *R* is the gas constant (=8.314 J K^−1^ mol^−1^), and *T* is the temperature in kelvin (=900 °C = 1173 K, see above). We show that both settling velocities are lower than the capture velocities for particle diameters up to ~80 µm, above which it may be interpreted as particles that would always be vented/never captured. This is roughly consistent with the PSD of the Cordón Caulle ash (Fig. 5). We note that these velocities are overestimates, as (1) turbulence often reduces the critical velocity^42^, (2) non-spherical particles have lower terminal velocities^43^, and (3) effects of surface roughness and build-up of captured particles, such that the critical “capturable” particle diameter is likely somewhat larger.

At peak velocities (100 ms^−1^), flows in the fracture pathways 0.01 m *< L* < 1 m are highly turbulent (7.14 × 10^3^ < Re < 7.14 × 10^5^). As the velocity decreases, flows in narrower pathways are more susceptible to transition to laminar flow. The threshold velocity at which the flow begins to transition into laminar flow occurs at ∼ 40 ms^−1^ for a 0.01 m-wide fracture, ∼ 4 ms^−1^ for a 0.1 m-wide fracture, or ∼ 0.4 ms^−1^ for a 1 m-wide fracture. As turbophoresis operates only in turbulent flows, below these velocities the delivery of carried ash particles to the fracture walls would likely be greatly reduced. Therefore, we infer that the majority of particle capture took place above these velocities. These velocities correspond to particles ∼12 µm and ∼60 µm in diameter, respectively, for 0.1 and 1 m-wide fracture pathways. The latter is in good agreement with the observed particle sizes in the HRN veneer interiors, suggesting most pathways were ∼1 m wide during active venting (Fig. 5). Should a pathway constrict, the threshold velocity for flow turbulence increases, corresponding to a decrease in the maximum diameter of particles that are likely to be captured upon impact.

**Fig. 6:**
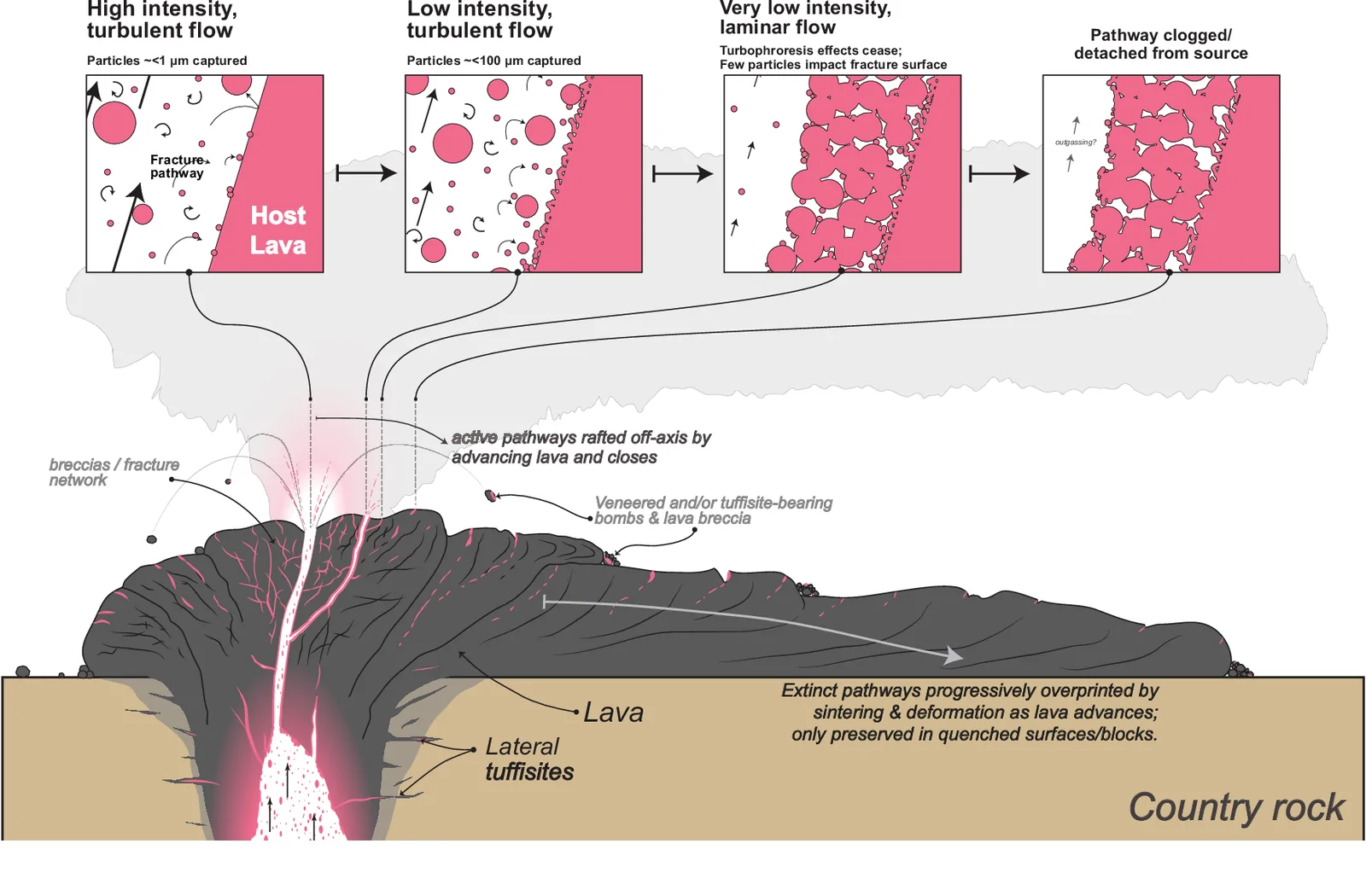
A generalized conceptual model for the transport of ash particles through lava, capture of ash particles on lava surfaces, and the formation of ash veneers. Overall, we suggest that the source of the exceptionally fine-grained ash found on veneer surfaces is likely to be sub-lava fragmental material, consistent with the “cryptic fragmentation” model [ref. ^15^]. Our model invokes microphysical processes for veneer emplacement: (1) turbulent flow delivers particles to the fracture surfaces via turbophoresis; (2) as flow velocity wanes, larger particles can be delivered to surfaces; (3) further waning of the velocity would shut-off particle delivery to the surface; and (4) fractures close, sealing up clogged fracture pathways. As the lava is advected out of the vent, veneers that were formed when that portion of the lava was atop the vent location, are also advected down-flow. Figure is not to scale.

Our interpretation is schematically represented in Fig. 6. We interpret the formation of a typical ash veneer, from its contact with host lava to the final deposition surface(s), to follow the following steps:

- The initial layer in contact with the host lava typically comprises small (∼1 µm) particles—captured during higher-intensity turbulent flows;
- Subsequent layer(s) of coarser (up to ∼100 µm) particles were captured during waning, lower-intensity, turbulent flows; smaller particles were still captured but are volumetrically less dominant.
- Inter-bedding of finer- and coarser- grained layers indicates fluctuating flows, either abruptly or gradually, over the lifespan of the venting pathway of ∼hours to days; some pathways were reactivated to high intensity, as evidenced by the presence of coherent rip-up clasts and erosional contacts;
- The flow eventually slowed such that it was no longer turbulent, and delivery of particles to the fracture walls via turbophoresis ceased. Very little material was then captured, though the pathway may have remained permeable.
- The surficial, ultra-fine particles may then be interpreted as turbophoretic capture during pulses of higher intensity flows within a constricted (<1 cm) pathway, or simply the surface expression of continuous capture of ultra-fine particles throughout the lifespan of the pathway.

During periods of high carrier phase velocity, turbophoresis preferentially concentrates the finest particles at fracture margins (c.f.^9^), so finer particle PSDs record energetic conditions. As flow velocity and turbulence wane, turbophoretic focusing weakens and larger particles, no longer fully entrained, become the dominant fraction deposited. Notably, this would result in inverted sequences relative to fluvial systems, where the dominant depositional process is (vertical) settling such that coarse material typically signals high transport competence, i.e., high velocities. Infrequent dune- or ripple-structures (e.g., Fig. 2b, c, h) may signal transient changes in the dominant depositional regime during the eruption, with turbulence- and sintering-governed mechanisms occasionally superimposed by vertical settling dynamics.

Our interpretations of these veneers therefore imply unsteady, pulsatory activity in silicic shallow conduits during hybrid eruptions, where individual pathways can be repeatedly activated, as inferred for tuffisite veins^21,44^. Additionally, relatively narrow (≲0.1 m) and/or short-lived fractures plausibly acted as pathways for ash venting but captured little to no material, preserving no textural evidence in the lava. Taking into consideration the close association of veneers and tuffisites (e.g., Fig. 2f), fracture pathways likely initiate as chaotic, tree-like networks^44^, of which the smaller branches progressively deactivate as ash particles are captured or as the host material expands by vesiculation. The final, planar pathway either fills up to form an internal tuffisite, or remains open, preserving the in situ depositional surface and forming lava veneers.

Estimating the lifespan of each veneer-pathway is not straightforward, as the material may have accumulated over multiple phases, with hiatae of unknown durations, during which the veneers—unlike internal tuffisite—could plausibly cool sufficiently to halt sintering due to influx of atmospheric air. Such hiatae may be common, considering that most HRN veneer materials are pink to red in color, indicating some duration of oxidation in the presence of atmospheric oxygen^45^. Accumulated material can also be removed by subsequent activity (e.g., Figs. 2g and 3e). Nevertheless, the low–moderate degree of sintering observed in most veneer samples can be leveraged to give a first-order estimate. We calculate the timescale *λ* (in seconds) needed for 1–100 µm particles to round by viscous relaxation^46,47^, by *λ* = *µr/*Γ [refs. ^47,48^],where *r* is the particle radius (in meters), *µ* is the melt viscosity (*µ* = 7.1×10^8^ Pa s—see above), and Γ is the interfacial tension between the droplet and the surrounding fluid (∼0.36 N m^−1^, [refs. ^9,49^]). At 900 °C, the relaxation timescale is ∼0.3–27 h—broadly overlapping the observed active timescales of clusters of fracture pathways^3,14,49^, estimated lifespans of tuffisites^20,21^, and the timescale required for effusing lava to replace the volume directly over the vent^6,50^. We note that this likely represents a lower-bound estimate for pathway lifespans, considering that erosion of deposited material appears to be common (e.g., Fig. 2g).

The presence of such lava-hosted ash veneers has implications for hybrid silicic eruptions, especially in the context of the cryptic fragmentation model^8^. Crucially, very-fine ash must have been produced in the conduit even during lava effusion, necessitating sustained primary fragmentation^8,9,15^. Considering that, for Cordón Caulle, the time-averaged mass eruption rate (MER) of the ash venting (~3.3 × 10^3^ kg s^−1^) is considerably lower than that of its lava effusion (3.9 × 10^4^ kg s^−1^), we suggest that this corroborates a clastogenic origin of the lava^8,15^. As a considerable portion of ash is captured between primary fragmentation and escape to atmosphere, vented PSDs should be systematically coarser than those generated at fragmentation^9,15^.

### Volcanic ash hazards during hybrid silicic eruptions

**Fig. 7:**
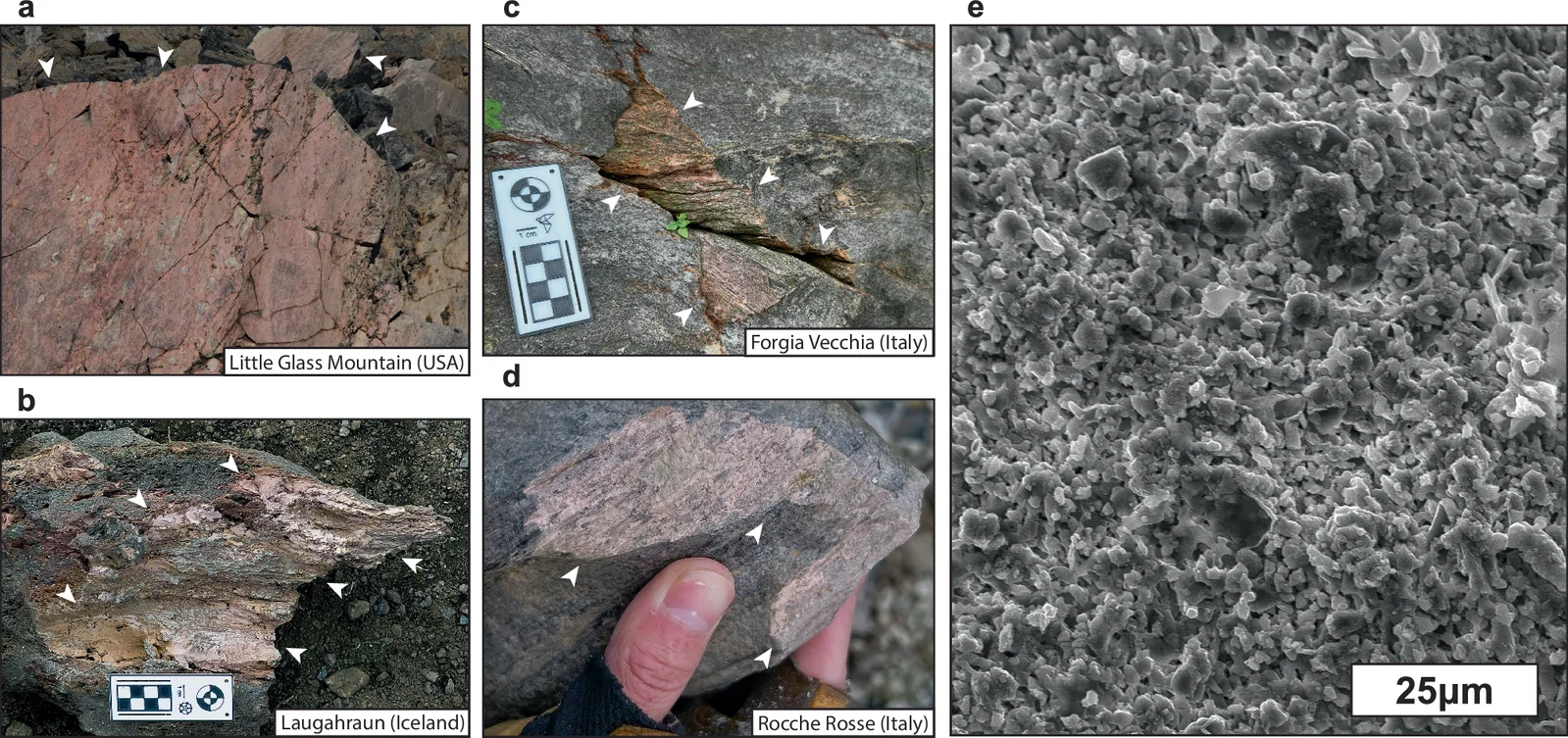
Ash veneers in silicic lavas elsewhere globally. Examples include: **a** Little Glass Mountain lava, Mono-Inyo, USA (same locality as in ref.^22^); **b** Laugahraun lava, Torfajökull, Iceland; **c** Forgia Vecchia lava, Lipari, Italy; **d** Rocche Rosse lava, Lipari, Italy. In all cases, white arrowheads indicate main veneer surface edges/extent. **e** A scanning electron microscopy image of a veneer surface collected from Forgia Vecchia, Lipari, Italy (see **c**).

With observations from Mono-Inyo, USA^9,22,51^ the 1960 and 2011–2012 lavas of Cordón Caulle, Chile^9^, the examined Holocene Torfajökull silicic lavas (this work), and the Holocene lavas Forgia Vecchia and Rocche Rosse^28^ on Lipari, Italy (this work; Fig. 7), we propose that ash venting is a common phenomenon which may well occur during any lava-producing silicic eruptions. Compared to the typically pulsatory activity at lava domes^52–55^, ash venting at lava-producing rhyolitic volcanoes is more likely to be near-continuous (as was the case during the 2011–2012 eruption of Cordón Caulle), possibly due to the constant advance of lava^50^ which prevents the buildup directly above the conduit that can intermittently suppress activity^53,56^.

The tephra output of the hybrid phase of silicic eruptions is poorly constrained, but assumed to be minor compared to the opening, high-intensity phase. Tephra mapping for the 2011–2012 Cordón Caulle eruption focused on the initial Plinian to sub-Plinian phase^11,30^, wherein the total mass attributed to the hybrid phase could not be assessed, only described as significantly lower than that produced on the last day of Plinian-subplinian activity (tephra layer K2, 2.8 ± 0.7 × 10^10^ kg)^11,30^.

We assess the total volume of tephra attributable to the hybrid phase of Cordón Caulle, from June 15th, 2011, to April 22nd, 2012—i.e., the full duration of hybrid activity. Over this period, plume heights averaged 2.6 km and showed an overall decreasing trend, but with considerable day-to-day variations. MERs were calculated from plume heights using an empirical relationship calibrated using 130 eruptions characterized in the IVESPA dataset^57^ (see “Methods”). We evaluate two end-member scenarios:

- Sporadic plumes: the reported plume heights represent daily maxima sustained for only 1 h each day, otherwise producing negligible output;
- Continuous plume: the reported plume heights were sustained for the entirety of the day (i.e., for 24 h).

**Fig. 8:**
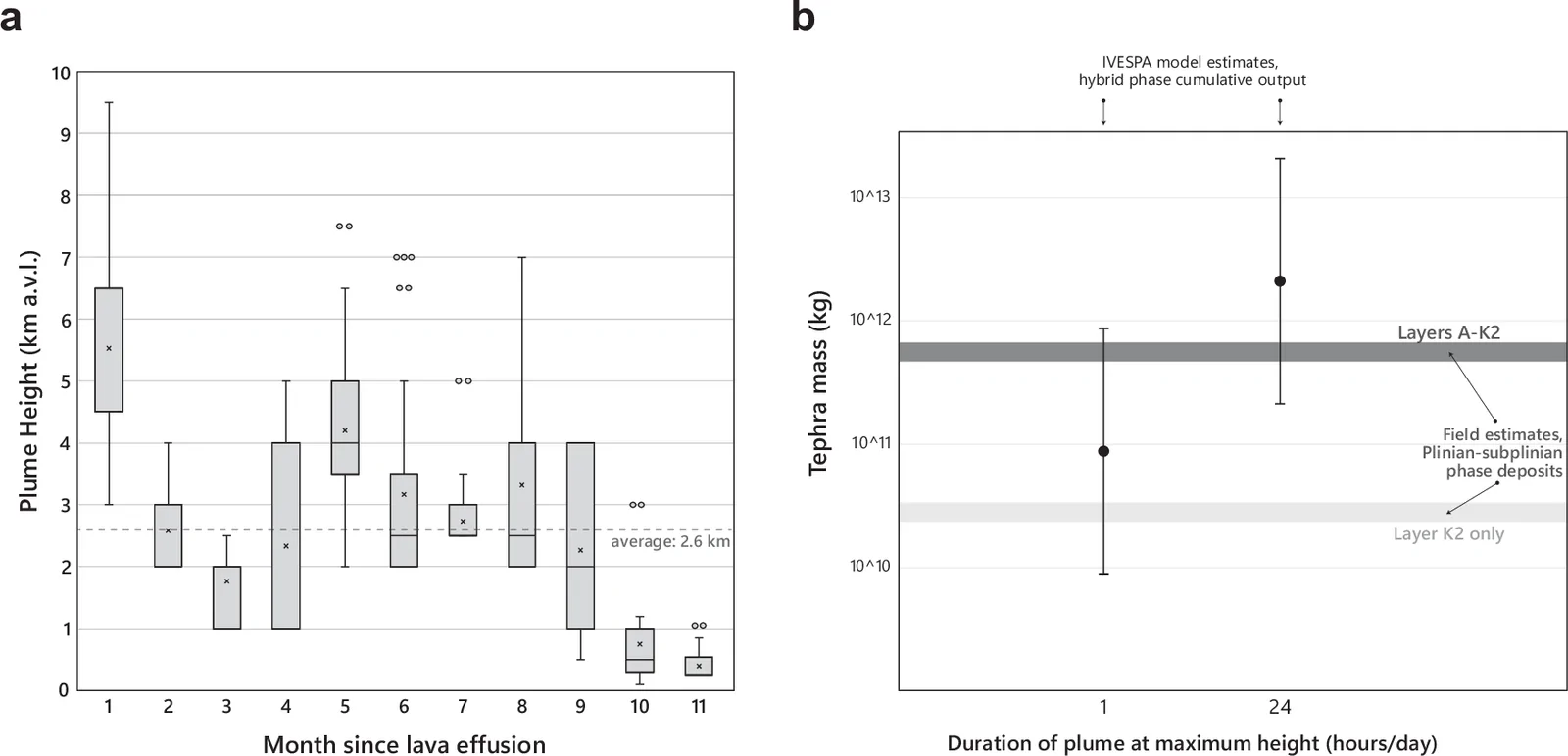
Inferred cryptic plume and tephra dynamics during lava effusion. **a** *Box-and-whisker plot of maximum plume heights for each 30 days of the hybrid eruption phase of Cordón Caulle*, showing the 25th-75th percentile ranges (filled boxes), mean values (crosses), median values (horizontal line in box, if different to mean), minima/maxima (whiskers), and outliers (circle dots). Overall average height (2.6 km) is also indicated as a horizontal dashed line. **b** *Estimated total mass of tephra produced during the ash venting phase of Cordón Caulle*, evaluated for the reported or interpolated daily maximum plume heights were sustained for only 1 h that day (the sporadic plume scenario), or the full day (i.e., 24 h; the continuous plume scenario). The main deposit of the Plinian-subplinian phase (layers A-K2) and its uppermost major layer (K2), as assessed from the field^31^, are indicated as horizontal bars, the heights of which indicate the uncertainties attributed by original workers.

Our best estimates for the total erupted mass for these scenarios are, respectively, 8.8 × 0^10^ kg (1-sigma uncertainty 1.5 × 10^10^—5.2 × 10^11^ kg) and 2.1 × 10^12^ kg (1-sigma uncertainty 3.5 ×10^11^–1.3 × 10^13^ kg). These are associated with time-averaged ash MERs of 3.3 × 10^3^ kg s^−1^ (1-sigma uncertainty 5.6×10^2^–1.9 × 10^4^ kg s^−1^) and 7.8 × 10^4^ kg s^−1^ (1-sigma uncertainty 1.3 × 10^4^ – 4.8 × 10^5^ kg s^−1^), respectively. Both scenarios produce comparable total output to the main deposit of the initial Plinian-sublinian phase of eruption (5.7 ± 1.0 × 10^11^ kg; [ref. ^11^]), and are considerably greater than that implied by field estimates^11,30^ (Fig. 8b), which may be attributed to a number of factors, including: (1) wider distribution of the fine ash dominated material^11^; (2) extensive syn-depositional remobilization^58,59^; (3) biased empirical MER-height relationship owing to HRN eruption source parameters (e.g., vent characteristics or duration) and plume dynamics very distinct from most of the hour-day long, primarily explosive eruptions documented in the IVESPA dataset used to calibrate the empirical relationship, and (4) general difficulty in reconstructing deposits of fine ash^60^.

A bulk volume of ∼0.4 km^3^ has been attributed to HRN tephra^24^, which we interpret to be primarily associated with its initial Plinian-subplinian phase. Considering the similar lava compositions, we assume HRN lavas (volume of ∼0.18 km^3^^24^) had a time-averaged output rate comparable to Cordón Caulle (2011–2012), at 17 m^3^ s^−1^ (i.e., ~3.9 × 10^4^ kg s^−1^^6^), which yields a lava effusion timescale of ∼170 days for HRN. We estimate the hybrid phase of HRN would have produced 4.8×10^10^ kg of tephra, assuming that ash venting is contemporaneous to lava effusion, and a similar time-averaged ash MER to the “sporadic plume” scenario of Cordón Caulle (i.e., 3.3 × 10^3^ kg s^−1^). Assuming the bulk density of HRN tephra is similar to that of Cordón Caulle, at 600 kg m^−3^^30^, this best estimate mass is equivalent to 0.08 km^3^ (~0.02 km^3^ dense rock equivalent), i.e., approximately 1/6 of its Plinian deposit, and comparable to estimates for the hybrid phase of Mono-Inyo volcanoes, USA^51^, or a VEI ~ 3 eruption by itself.

While the distal tephra stratigraphy of HRN is fairly well studied in Iceland^24^, there is no confirmed evidence of the hybrid-phase HRN ash in distal deposits. We suggest that a combination of factors likely contribute to the absence of this tephra, including: (1) its gradual deposition at low MER over an extended time period, making syn-depositional remobilization more influential than for rapidly-emplaced tephra; (2) the ash plume will have likely repeatedly changed in spreading direction with changing wind directions through time, increasing overall dispersion and thus reducing preservation potential, and (3) given the long duration and location of eruption, the deposition is likely to have been affected by snow fall and subsequent thawing, which will further reduce its tephra preservation potential. Due to large uncertainties with preservation potential from past eruptions, especially those with lower magnitudes, it may also be the case that the deposit from this phase has been overlooked^61^. We suggest that for comparable past eruptions, their hybrid phases would have similarly produced a sizeable quantity of tephra that is not preserved and/or accurately assessable, hence absent from the record as a unique and identifiable discrete layer.

Importantly for the consideration of hazards, plume heights during the eruption of Cordón Caulle often exceeded its average of 2.6 km, even months after the hybrid phase began, with plume heights reaching 7 km as late as month eight of the eruption (Fig. 8a). Such unpredictably fluctuating plume heights contributed to the re-closure of Bariloche airport (Argentina), ∼100 km east of Cordón Caulle, days after its reopening in January 2012 (via BBC: https://www.bbc.co.uk/news/world-latin-america-16587541). We suggest that efforts to assess the hazard of the hybrid phase of silicic eruptions must consider such scenarios of short-timescale fluctuations. Seismic monitoring may help inform hazard assessment during an ongoing hybrid eruption^6,62^.

In addition to modifying the deposit, remobilization of vented ash produces further, longer-lived hazards^63^ to public health^59,64^ and infrastructure. Wind remobilization of ash from Cordón Caulle occurred during its hybrid phase and continued to pose lasting hazards to communities >200 km away for >8 years after the complete cessation of the eruption^59,65^. A significant number of Icelandic population centers, including the capital Reykjavík and its international airport, are located <200 km from Torfajökull, with Vík and Selfoss located <100 km away. Remobilization of volcanic ash in Iceland is further complicated by uncertainties from the source locations and often-severe weather conditions^66^. Several major re-suspension events reached Icelandic population centers—including Reykjavík—following the 2010 eruption of Eyjafjallajökull and 2011 eruption of Grímsvötn^66–68^. The hybrid phase of an HRN-like rhyolitic eruption within Torfajökull could potentially produce comparable volumes of tephra to Eyjafjallajökull 2010 eruption (0.27 km^3^, ref. ^10^) in addition to its Plinian-phase deposits—highlighting the hazard potential of such remobilization effects.

More broadly, we urge that it is prudent to assess prolonged, near-continuous ash venting for all lava-producing silicic systems worldwide, even if an associated deposit for such has not been identified. We propose that the estimated effusion timescale of lavas can be used as a first-degree approximation to assess the duration and total output of these sustained but poorly documented ash emissions.

## Methods

### 2D Particle size analysis

Scanning Electron Microscope (SEM) images were collected at Lancaster University, with a JEOL JSM-7800F SEM. Carbon coating was used for samples from which EDS data were collected, while gold coating was used for imaging-only samples for better resolution. We manually traced distinguishable particles on SEM images of veneer surfaces, which were then analyzed via ImageJ. A range of 2500–5000× (pixel resolutions of 0.01–0.02 µm) magnifications were used, consistent with past study^9^. A total of 2304 particles were traced and analyzed, using SEM imagery from 3 samples, with each sample having a minimum of 463 analyzed particles (Supplementary Fig. 3).

Additionally, we traced visible particles on optical photomicrographs, imaged from standard petrographic thin sections cut ∼orthogonal to the veneer surface. Photomicrographs were collected using transmitted plane-polarized light and a 20× objective, achieving a pixel resolution of 0.2 µm. Particles whose minor axis is <5 µm (i.e., <25 pixels) were discarded to mitigate uncertainties in the tracing process. A total of 2639 particles were traced and analyzed in 4 groups, across 2 image mosaics (each consisting of ∼10 photomicrographs; see Supplementary Fig. 4). For the purposes in this paper, considering their limitations^69^, we use the 2D PSDs to inform the range of particle sizes observed in the samples, rather than more detailed analyses of the distributions. All particle size data generated in this study is provided as Supplementary Data S1.

### Laser diffractometry

During the 2011–2012 eruption of Cordón Caulle, ash was deposited on tents overnight on Jan 4th, 2012, a sample of which was collected^3^. This sampling location was 3.8 km NNW of the vent. This sample was sieved to <2 mm, treated with hydrogen peroxide to remove any contaminating organic material, then treated with a 10% sodium hexametaphosphate solution to suppress flocculation. The treated sample was then physically re-homogenized. Approximately 1 g of material was analyzed using a Beckman Coulter LS-13320 laser diffraction particle size analyzer at the Lancaster Environment Centre, Lancaster University. Water was used as the dispersant/carrier fluid, and a 30 s sonication applied prior to analysis. The instrument then analyzed the sample three times in succession, the average of which was taken. A particle size range of 0.04–2000 µm was achieved. All particle size data generated in this study is provided as Supplementary Data S1.

### Mass eruption rate and cumulative tephra output

We collated reported plume heights^11,13^ for the 2011–2012 eruption of Cordón Caulle, over the duration of June 15th, 2011 when the lava effusion was inferred to begin^6^, to April 22nd, 2012, the last report of an ash-laden plume. We applied the IVESPA v1.0 -calibrated, empirical power-law model^57^ to calculate MER from these plume heights. The model is log_10_(MER) = 2.83 + 3.54 × Log_10_(*H*_top_), where MER is the MER given in kg s^−1^, and *H*_top_ is the plume top height in km above vent. Model uncertainties were provided at 1-sigma confidence interval using the look-up table provided in ref. ^56^. When applying the relationship to Cordón Caulle, we assume the reported plume heights, where unspecified, refer to the top heights (rather than spreading heights). All plume heights in this work refer to the top height, above vent level.

Plume heights were not reported for 136 days out of 313 days of the hybrid phase eruption, mostly noted due to adverse weather conditions. For these days, we take the minimum reported plume heights within 15 days prior. Our strategy here aims to produce an overall conservative estimate for both scenarios we evaluated. Note that for strongly wind-affected plumes, MER derived by empirical modeling are potentially lower by ≲1 order of magnitude, as was discussed for the early phase of the 2011–2012 eruption of Cordón Caulle^11^, which we do not explicitly account for. All plume heights used this study and the associated MER values are provided as Supplementary Data S2.

## Supplementary information

Supplementary Information

Description of Additional Supplementary File

Dataset 1

Dataset 2

Transparent Peer Review file

## Data Availability

All data generated in this study are provided as Supplementary Information and Data, and have been deposited at Zenodo (https://doi.org/10.5281/zenodo.18173806).
