# Supplementary material for: Sustained ash emission during lava effusion is a hidden volcanic hazard of silicic eruptions
Source: Nat Commun. 2026 Aug 8;17:9539. doi: 10.1038/s41467-026-76464-w (PMC13547391; doi:10.1038/s41467-026-76464-w)
Supplement: Supplementary file 1 [file 41467_2026_76464_MOESM1_ESM.pdf]

Sustained ash emission during lava effusion is a hidden volcanic hazard of silicic eruptions

Supplementary Material

Jingwei Zhang[1*], Hugh Tuffen[1], Fabian B. Wadsworth[2], Jamie I. Farquharson[3], Holly E. Unwin[4], Alastair G. E. Hodgetts[5], Thomas J. Aubry[6], Maciej W. Farbicki[1], Dave McGarvie[1], Daníel Freyr Sigurbjargarson[7]

[1*]Lancaster Environment Centre, Lancaster University, Lancaster, LA1 4YQ, UK.
[2]Department of Earth and Environmental Sciences, Ludwig-Maximilians-Universität München, Munich, 80333, Germany.
[3]Institute for Research Administration, Niigata University, Ikarashi 2-8050, Nishi-ku, Niigata, 950-2181, Japan.
[4]British Geological Survey, Nicker Hill, Keyworth, NG12 5GG,UK.
[5]School of GeoSciences, University of Edinburgh, King's Buildings, Edinburgh, EH9 3FE,UK.
[6] Department of Earth Sciences, University of Oxford, S Parks Rd, Oxford, OX1 3AN,UK.
[7] Nature Conservation Agency of Iceland, Austurvegur 4, 860 Hvolsvöllur, Iceland.

*contact: j.zhang77@lancaster.ac.uk

This Supplementary Material includes the following items:

1. The results of measuring the major element compositions of the key samples, by Electron Dispersive X-ray spectroscopy via a Scanning Electron Microscope.
2. The calibration data and visualization for the sticking model presented in the main text.
3. Scanning Electron Microscope (SEM) images from which grainsize distributions were measured.
4. Transmitted light microscopy images from which grainsize distributions were measured.

Our particle size measurements, both from image analyses (parts 3 & 4, this document) and laser diffractometry, are included as a separate Excel spreadsheet.

Our compilation of plume heights of the 2011-2012 Cordon Caulle eruption, as well as calculations thereof, are included as a separate Excel spreadsheet.

1. Major element compositions

We used Energy-dispersive X-ray spectroscopy (EDX) to fingerprint the major element compositions of 3 veneer-bearing HRN samples. Analyses were carried out on carbon-coated, uncovered petrographic thin sections.

The data pertaining to these compositions is given here in Table S1 and visualized on a total-alkalis vs. silica (TAS) diagram along with previously published data (Fig. S1; refs).

Supplementary Table 1. Major element compositions of HRN veneer-bearing lavas. All compositions are given in wt.%, normalized to 100%. b.d. = below detection.

ID	type	SiO2	TiO2	Al2O3	FeOT	MnO	MgO	CaO	Na2O	K2O	P2O5
65	lava	66.8	0.8	15.7	4.7	0.1	0.3	1.8	6.3	3.5	b.d.
65	veneer	72.0	0.6	15.3	1.1	b.d.	0.2	0.5	5.9	4.4	b.d.
108	lava	72.5	b.d.	16.1	0.3	b.d.	b.d.	0.6	7.3	3.2	b.d.
108	veneer	71.4	0.2	15.8	0.7	b.d.	0.3	0.7	7.7	3.2	b.d.
108	veneer	71.7	b.d.	15.9	0.8	b.d.	0.3	0.8	7.1	3.4	b.d.
108	veneer	71.6	b.d.	16.5	0.6	b.d.	0.4	0.7	6.8	3.4	b.d.
108	veneer	72.1	b.d.	16.4	0.5	b.d.	b.d.	0.7	6.6	3.7	b.d.
115	lava	71.6	b.d.	17.0	0.5	b.d.	b.d.	0.8	7.3	2.8	b.d.
115	veneer	67.8	b.d.	2b.d.	0.1	b.d.	2.3	b.d.	9.2	0.6	b.d.
115	veneer	72.4	b.d.	16.8	b.d.	b.d.	b.d.	0.5	7.8	2.5	b.d.
124	lava	71.7	b.d.	16.6	b.d.	b.d.	0.5	b.d.	8.7	2.5	b.d.
124	lava	72.3	b.d.	16.7	b.d.	b.d.	b.d.	b.d.	8.7	2.3	b.d.
124	lava	73.0	b.d.	15.7	0.8	b.d.	b.d.	b.d.	6.8	3.7	b.d.
124	lava	73.2	b.d.	16.0	b.d.	b.d.	b.d.	b.d.	8.2	2.6	b.d.
124	lava	73.4	b.d.	15.5	0.8	b.d.	b.d.	b.d.	6.5	3.8	b.d.
124	veneer	72.5	b.d.	16.4	0.2	b.d.	b.d.	b.d.	8.2	2.7	b.d.
124	veneer	72.7	b.d.	15.4	0.6	b.d.	0.6	0.7	6.6	3.4	b.d.
124	veneer	73.0	b.d.	16.3	0.3	b.d.	b.d.	b.d.	6.9	3.5	b.d.
107A	lava	68.7	0.7	19.6	0.7	b.d.	0.8	0.8	6.6	2.1	b.d.
107A	veneer	71.4	b.d.	18.2	b.d.	b.d.	b.d.	0.6	7.8	2.0	b.d.

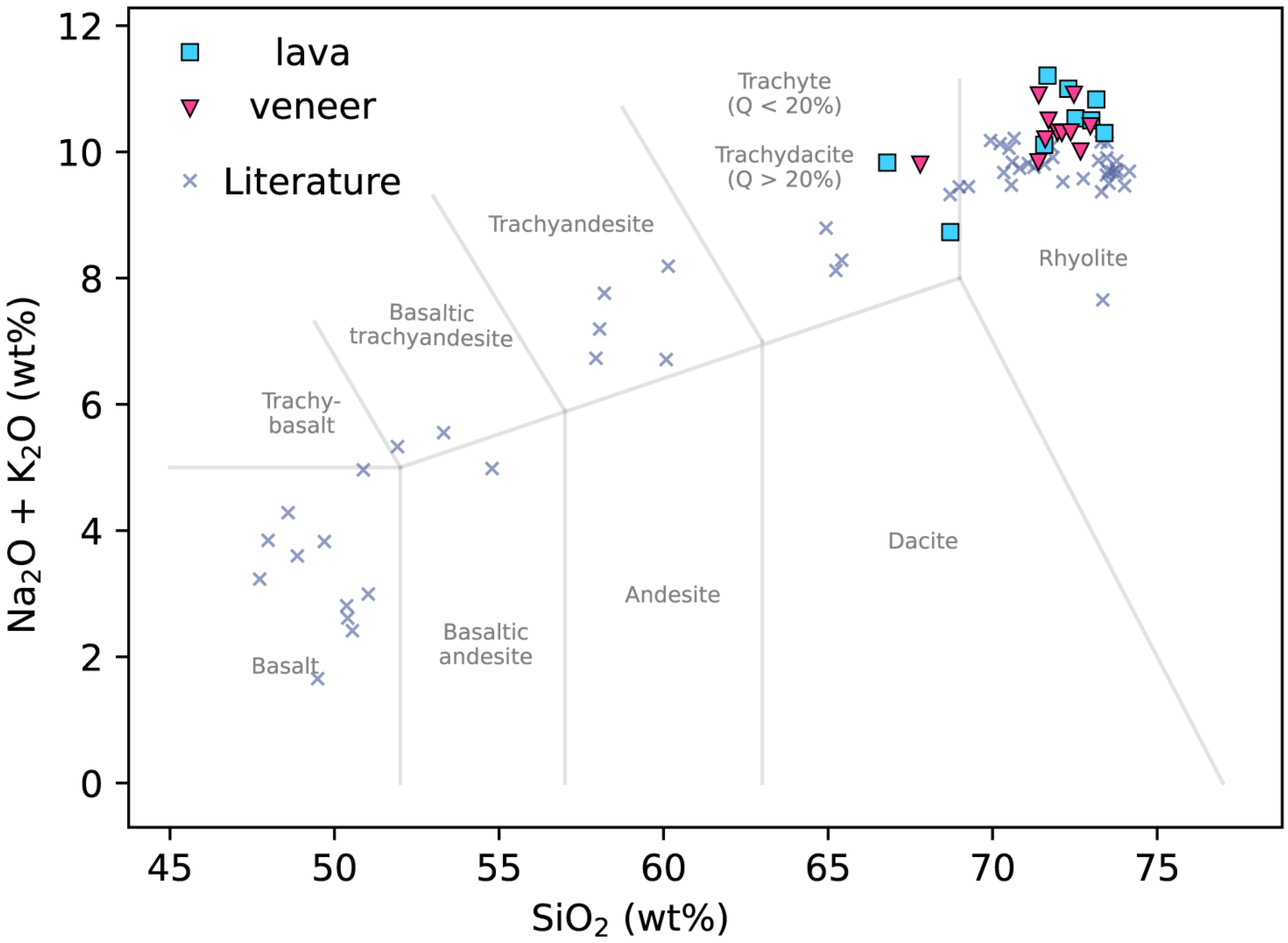

Supplementary Figure 2. The total-alkali vs. silica diagram for HRN compositions. Shown here are the compositions of Hrafntinnuhraun ash veneers and their host lava obtained in this study, alongside relevant compositions from literature (McGarvie et. al, 1989, Reference [25] in the main manuscript).

2. **Scanning Electron Microscope (SEM) images, from which grainsize distributions were measured.**

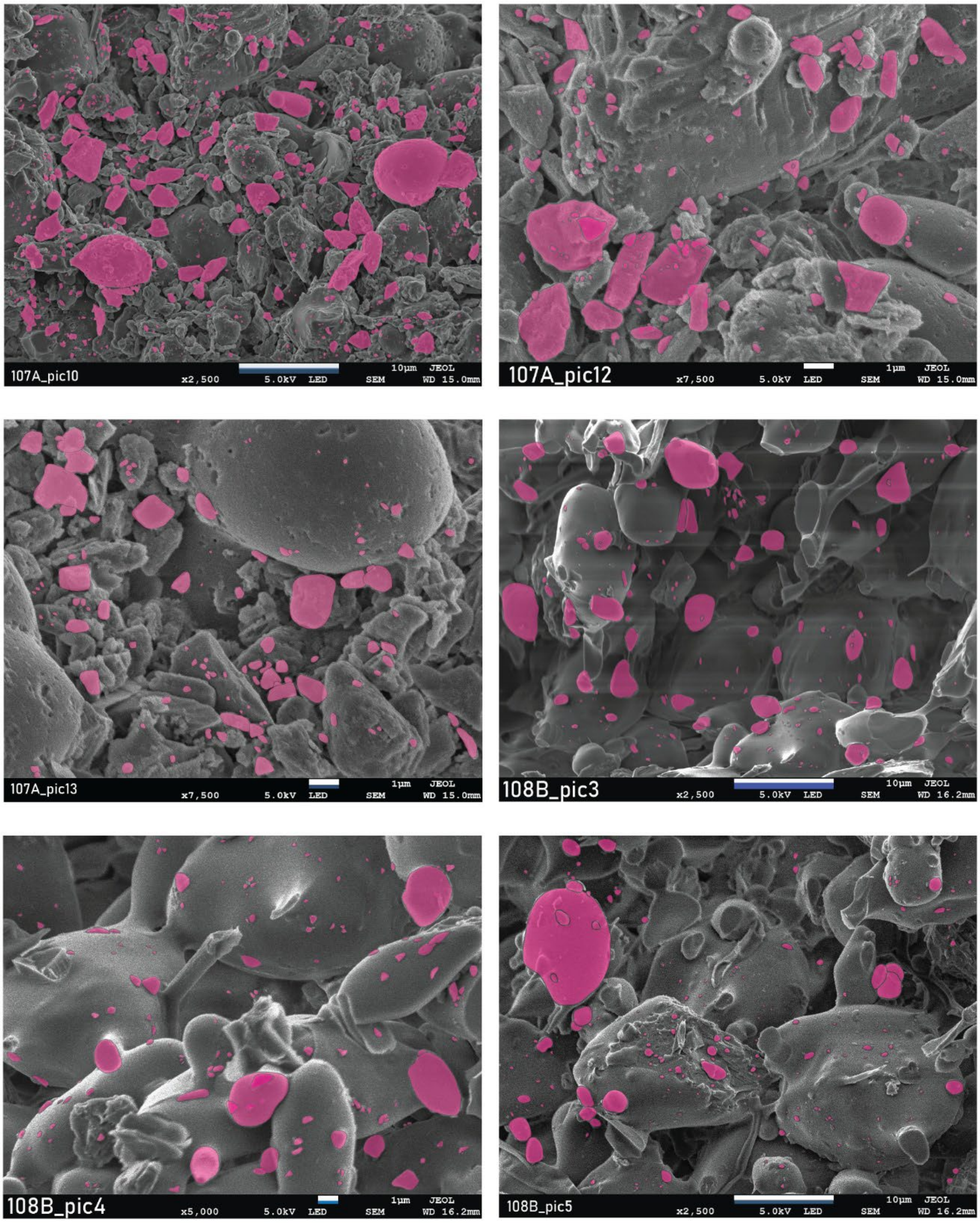

(continued)

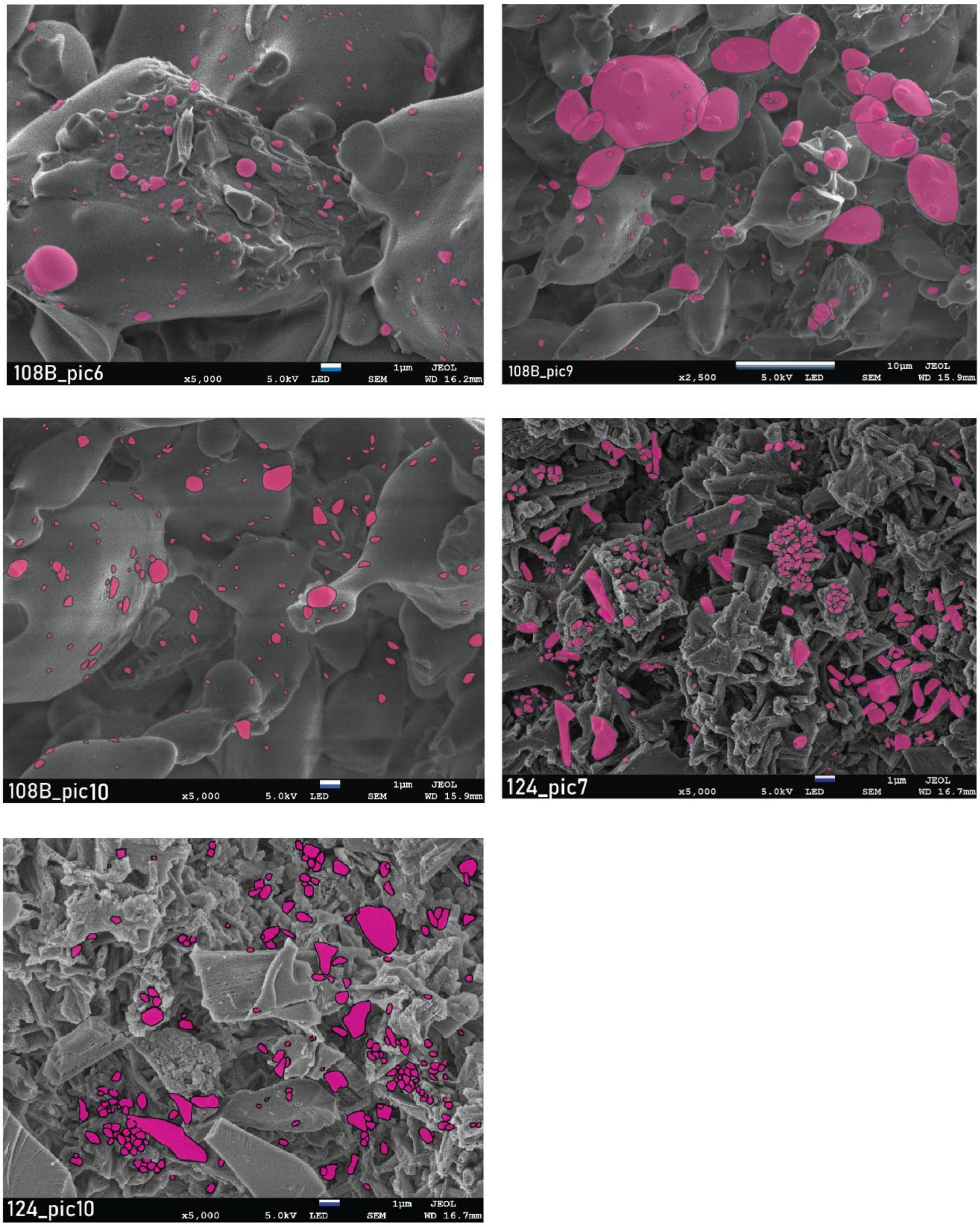

Supplementary Figure 3. SEM images of the *in situ* HRN veneer surfaces, from which PSDs were derived in this study. Pink overlays show the particles that were traced and used in the subsequent analysis. The methodology follows that of Farquharson et al., 2022 (Reference [9] in the main manuscript; doi: 10.1038/s41467-022-32522-7).

3. **Transmitted light microscopy images from which grainsize distributions were measured.**

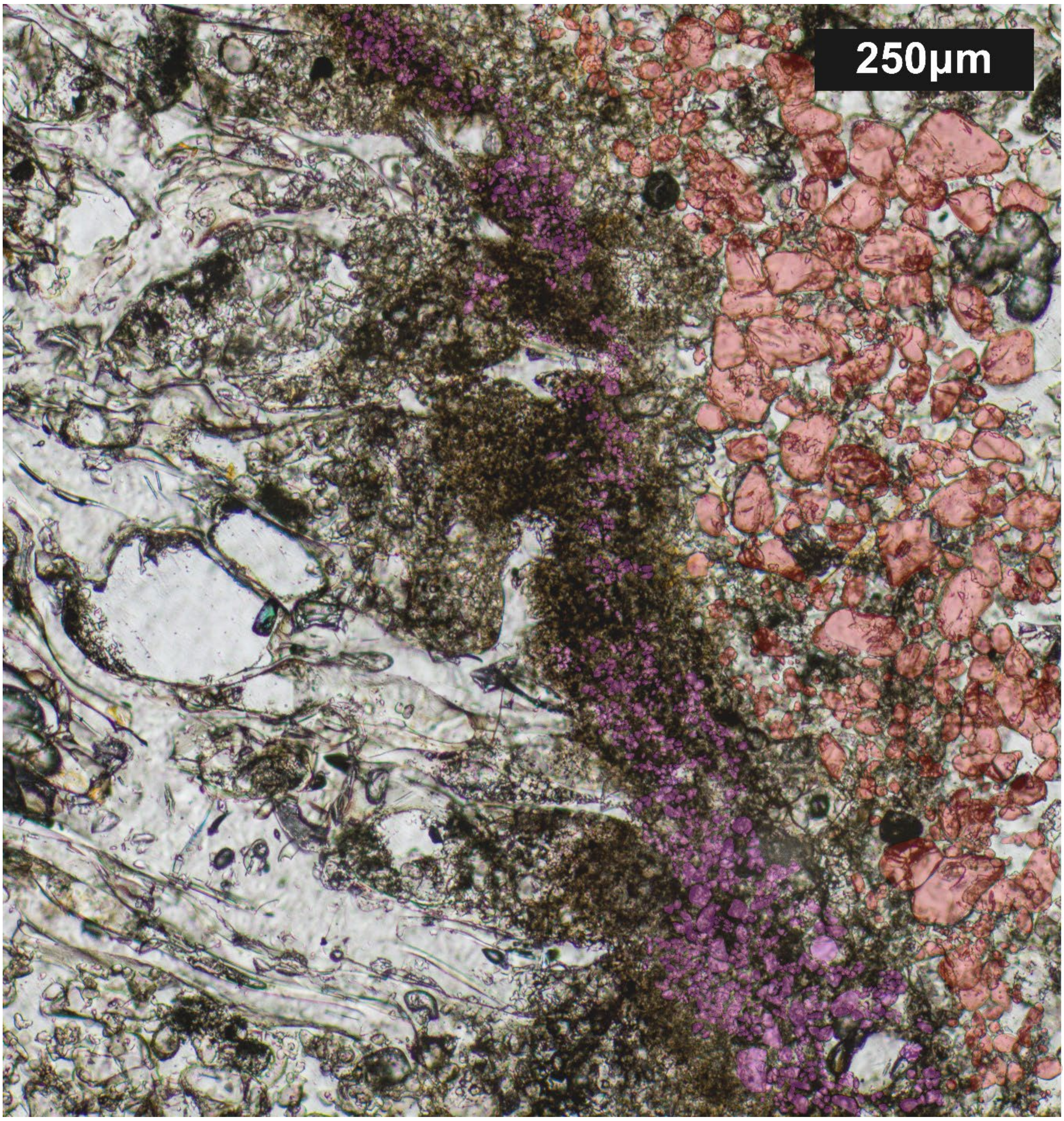

(continued)

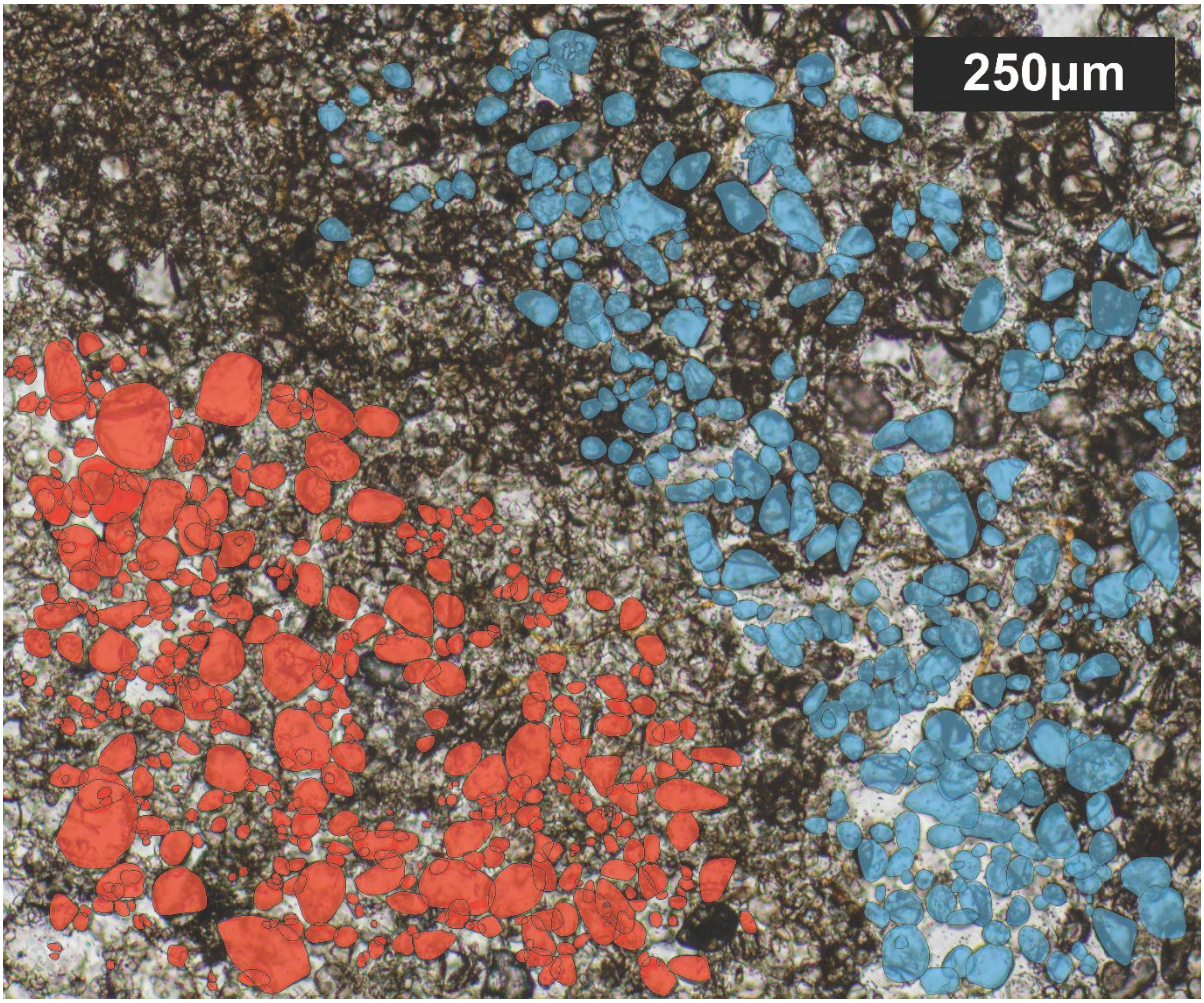

Supplementary Figure 4. Transmitted-light photomicrograph mosaics of the HRN veneer thin section, from which PSDs were derived in this study. Color overlays show the particles that were traced, with each of the four colors corresponding to a band with which particles are grouped in the subsequent analysis.

4. Calibrating a sticking model to determine a critical sticking viscosity

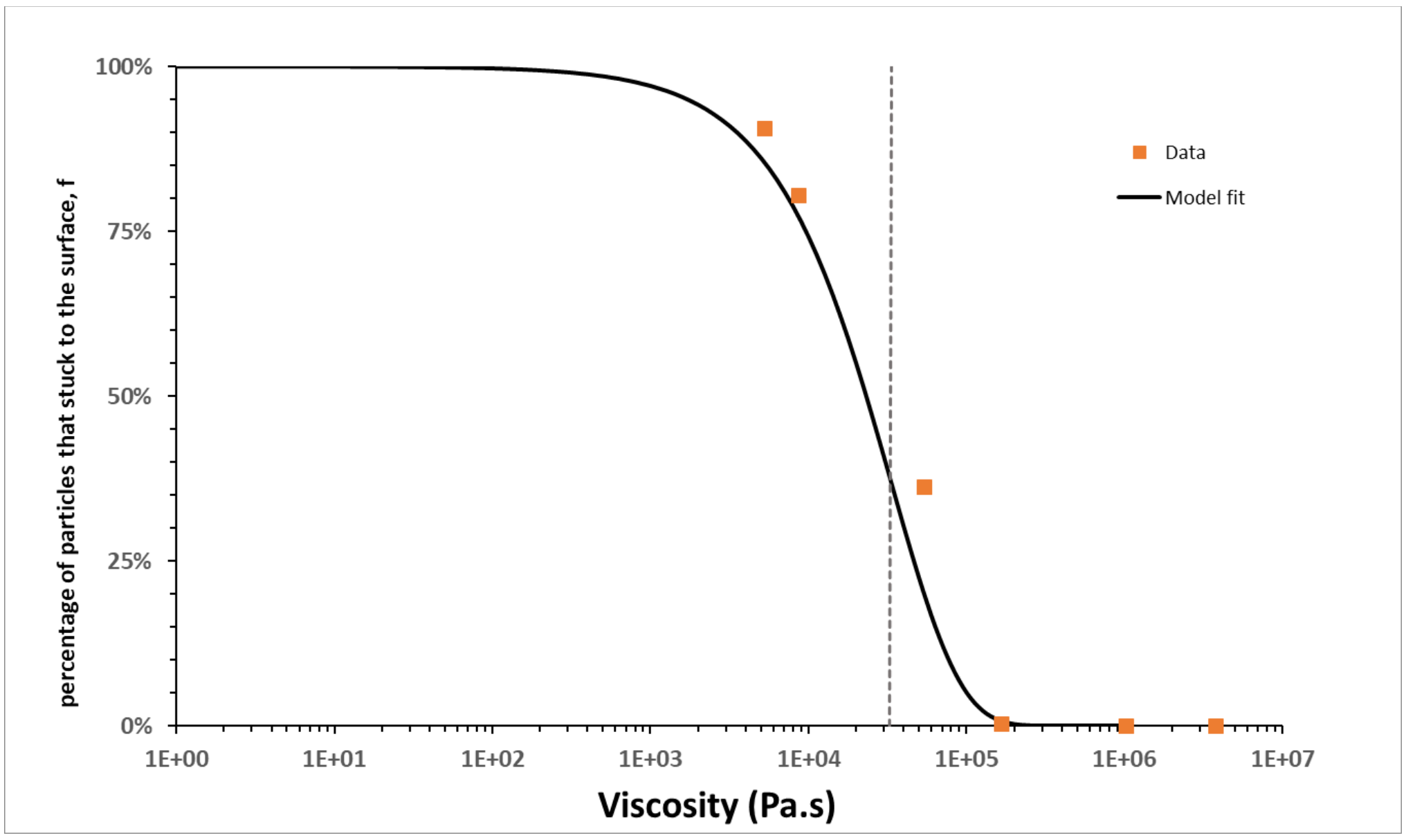

Supplementary Figure 4. The critical sticking viscosity. The data are from Srinivasachar et al. (1990) where $f = 100\%$ refers to all of the particles sticking to the surface, whereas $f = 0\%$ refers to none of the particles sticking to the surface. The viscosity-temperature relationship is $\log_{10} \mu = 12.047/T - 6.098$. We mark the critical sticking viscosity with a vertical dashed line.

The sticking model presented in the main text predicts the critical viscosity above which particles will bounce and below which particles will stick to surfaces. In order to determine this, we used datasets from Srinivasachar et al., 1990 (Reference [38] in the main manuscript), in which soda-lime-silica glass spheres were pre-heated and dropped onto surfaces. In the original study, the authors determine a critical sticking temperature range (their Fig. 3), above which 100% of the particles stuck to the surface and below which 0% of the particles stuck to the surface.

Here, we did two things to analyze and generalize these data.

- First, we used their stated viscosity-temperature relationship to convert their sticking as a function of temperature to a notion of sticking as a function of viscosity. That viscosity relationship is $\log_{10} \mu = 12.047/T - 6.098$ where T is in kelvin.
- Second, we fit a continuous curve to their sticking data (which is cast as the number percentage of all particles that stuck; in the main text this would be f multiplied by 100, where f is the sticking fraction).

As stated in the main text, we fit the function $f = \exp(-\mu/\mu_c)$, and we use a least squares regression algorithm to determine $\mu_c = 3.341 \times 10^4$ Pa.s (see Fig. S2).
