# Supplementary material for: Sustained ash emission during lava effusion is a hidden volcanic hazard of silicic eruptions
Source: Nat Commun. 2026 Aug 8;17:9539. doi: 10.1038/s41467-026-76464-w (PMC13547391; doi:10.1038/s41467-026-76464-w)
Supplement: Supplementary file 2 [file 41467_2026_76464_MOESM2_ESM.pdf]

Description of the Additional Supplementary Files

Supplementary dataset 1: A compilation of all particle size data used in this study, including: (1) collected by laser diffraction, (2) collected by image analysis of transmitted light photomicrographs, (3) image analysis of scanning electron microscopy images, (4) from the dataset of Farquharson et al. 2022.

Supplementary dataset 2: A spreadsheet containing (a) the compilation of plume heights, their data sources (including those interpolated in this study, see Methods), associated mass eruption rates (MERs) calculated using the IVESPA v1.0 -calibrated power-law model (including ± 1-sigma range estimates), and the cumulative outputs from both "sporadic" and "continuous" scenarios.
