# Supplementary material for: Sustained ash emission during lava effusion is a hidden volcanic hazard of silicic eruptions
Source: Nat Commun. 2026 Aug 8;17:9539. doi: 10.1038/s41467-026-76464-w (PMC13547391; doi:10.1038/s41467-026-76464-w)
Supplement: Supplementary file 3 [file 41467_2026_76464_MOESM5_ESM.pdf]

nature portfolio

Peer Review File

Sustained ash emission during lava effusion is a hidden volcanic hazard of silicic eruptions

Corresponding Author: Mr Jingwei Zhang

This file contains all reviewer reports in order by version, followed by all author rebuttals in order by version.

Version 0:

Reviewer comments:

Reviewer #1

(Remarks to the Author)
Sustained ash emission during lava eruption is a hidden volcanic hazard of silicic eruptions
Zhang et al.

Review comments by:
Magnús T. Gudmundsson, 15 February 2026

This manuscript presents in many ways a new insight into possible style of silicic eruptions where ash emission may accompany/be a major part of what have been considered predominantly effusive eruptions. The Cordon Caulle eruption in Chile in 2011-2012 is used as an example of such events. The possible occurrence of this type of behavior for the ~877 AD Hrafntinnuhraun eruption (the silicic part of the largely basaltic explosive Vatnaöldur eruption) is advocated. The explosive tephra "eruption" is considered to have occurred through fractures in the high viscosity lava directly above the vent. Detailed field descriptions of the evidence for veneers coating fractures in the lava and a conceptual model are presented. Estimates of likely tephra production during these assumed hybrid events (effusive + explosive) are presented and the amount of explosively transported tephra is presented, indicating that its volume, erupted throughout the lifetime of the eruption (of order 150-200 days), may have been similar to the volume of the lava formed in the same event.

This idea is most interesting and the results as well. It is advocated that explosive activity/tephra production (apparently predominantly fine ash) occurring during the effusive phases of these silicic eruptions is a hazard that has not been properly identified before.

Although this is highly interesting, it has to be said that there is little empirical evidence for such a significant explosive phase occurring at the same time as the silicic lava eruption at the location studied (Hrafntinnuhraun). In known explosive eruptions of moderate/low intensity, the dispersive power is much less than in more powerful eruptions. As a consequence, tephra tends to pile up in the vicinity of the vents. Also, if the volumetric flow rate of tephra was of the same order of magnitude as that of the lava (~half a cubic kilometer), a sizeable tephra layer would be found in the region, as well as prominent craters. If deposited mostly locally (due to low intensity) the latter would probably be dominant. Moreover, if similar to 0.5 km3 of tephra was erupted over 180 days (density 600 kg/m3) the average tephra MER would have been similar to 200,000 kg/s. This would have been almost twice the average MER in the 39-day long Eyjafjallajökull eruption in 2010 (3.8 x 10pow11 kg ~ 120,000 kg/s). The 2010 Eyjafjallajökull tephra layer is well preserved in soil in a large region extending tens of kilometers from the volcano. Preservation potential in the 9th century AD would have been considerably better than today, when birch/tree cover was dominant on the lowlands.

For the 877 eruption a silicic tephra layer is found underneath the much larger basaltic layer formed at the long fissure to the north of Torfajökull (see maps and descriptions in Larsen, 1984). This shows that at least the main explosive silicic phase was not long-lived and occurred at the beginning of the main explosive phase. Hence, the authors should be more careful in their assertions on the relevance of co-effusive explosive activity for this eruption. The empirical data seem to indicate that any co-lava effusion explosive activity was orders of magnitude smaller – airphotos (e.g. www.map.is) and field observations suggest that there is relatively little tephra covering Hrafntinnuhraun. In contrast, on air photos of Cordon Caulle (e.g. google-earth), tephra is quite prominent, apparently covering parts of the lava.

The above is to an extent discussed in line 394-404. However, if the volume produced was similar to that suggested, parts of it should be visible in some of the hundreds of tephra sections studied over several decades in Iceland. If this occurred over extended period, the tephra should be on top of the 877 basaltic tephra formed in the main phase of this eruption. But it is not observed. I therefore suggest that the manuscript should be updated where the nuances/empirical evidence discussed above are fully taken into account.

Minor comments:
79-~100: Consider the beginning phases of the Hekla eruptions - they are typically mixed eruptions with explosive phase being dominant to begin with, while effusive activity takes over after the first few hours – however, some tephra is at times deposited for weeks (e.g. 1947).

115: According to the geological map of Iceland, Hrafntinnusker is considered ~9000 years old. See: https://arcgisserver.isor.is/?lon=-19.29484&lat=63.96733&zoom=12&layers%5B%5D=satellite&layers%5B%5D=geologyDetailed

116: This eruption has been dated to 1477 AD – see e.g. www.icelandicvolcanos.is - look upp Barðarbunga – volcano information – 11. Lagest known eruption. This has not been properly documented in peer-reviewed papers but is based on a written letter from Eyjafjörður in North Iceland in 1477, describing the tephra fallout. Recorded in e.g. Náttúruvá á Íslandi, Eldgos og jarðskjálftar, p. 257, where a citation to the source is given.

258: 1.341 x 10 4 Pa - four significant digits? Given the uncertainties, 1.3 is more appropriate than 1.341.

268; 7.09 more appropriate to use 7.1 (see above – significant digits)

415-419: 0.48 km3 - if this value is correct, where is the tephra? Hundreds of tephra layers, most smaller than this volume are preserved in the soils from the Holocene time in Iceland. This value is larger than that for the Eyjafjallajökull eruption of 2010…..

440-444: Cite sources for Eyjafjallajökull tephra volume. The number given comes from Gudmundsson et al. 2012. Scientific Reports https://www.nature.com/articles/srep00572

Figure 1: Not a major issue, but the paper would be stronger if a proper map showing the location and context of the eruption site/Hrafntinnuhraun would be shown. The current map is not very informative/showing the context + the link of the eruption with the main eruption site of Vatnaöldur.

657: The reference is incomplete – should be:
Larsen, G. 1984. Recent volcanic history of the Veidivötn fissure swarm, south Iceland – an approach to volcanic risk assessment. Journal of Volcanology and Geothermal Research, 22, 33-58.

Reviewer #2

(Remarks to the Author)
Review of: Sustained ash emission during lava effusion is a hidden volcanic hazard of silicic volcanic eruptions
Authors: Zhang et al.
Reviewer: Benjamin J. Andrews, Smithsonian Global Volcanism Program

The authors describe fine grained veneer and tuffisite deposits on surfaces and fractures from the Hrafntinnuhraun flow in Iceland. They interpret those deposits are recording transport of fine-grained ash through the fractures with finer grained material sintering on the margins during periods of high velocity gas flow, and particle size increasing as the gas flow wanes. They estimate that individual fractures likely remain open for many hours to days, suggesting that continuous emission of ash can occur from silicic lava flows. Through analogy with the Cordon Caulle eruption, the authors estimate that about 0.48 km3 of fine ash was erupted during the ~6 month eruption timescale of the lava. Finally, they point out that this type of eruption could produce sustained and substantial hazards to local and regional communities, but that this hazard is largely unrecognized.

There are several big ideas that the paper raises. First, the authors bring in the concept of turbophoresis – whereby particles migrate to regions of lower turbulence intensity – in the interpretation of the deposits. Second, the authors explain how and why particles should stick and sinter within the fractures versus why they escape – importantly, they quantify this process to explain the granulometry of the deposits in the fractures/veneers and why observed “extra vent” deposits at Cordon Caulle are ~100um. Together, the turbophoresis and stick-bounce criteria that the authors develop have interesting implications for thinking about ash transport through and deposition within fractures, and for volcanic conduit processes in general.

There are two or three areas in which the paper can be strengthened. First, the authors make a big deal out of turbophoresis, but this concept or process could be explored in a little more detail. Specifically, what types of turbulence intensities (and cross-fracture gradients in turbulence) might we expect to ee in these fractures? The turbulent velocity distributions, and the timescales over which those fluctuations occur, are probably more important for determining deposition of the particles than the mean flow velocities. Second, there is a minimum flow velocity required to transport particles of a given size and density, and this velocity is almost certainly greater than the maximum “sticking” velocity of the larger particles. As a result, once

particles become sufficiently large, they basically cannot stick onto the fracture margins. This mobilization velocity is basically the particle settling or fall velocity, and could be plotted on Figure 5 as a line that increases with size (rather than decreasing like the sticking velocity) to show a maximum particle size. Finally, the paper suggests that during intervals of high gas velocity, only small particles are deposited, whereas larger particles get deposited as velocity wanes. This is a big deal as it is more or less the opposite of fluvial processes (large particles indicate high fluid velocities, and fine muds indicate low-velocity or slack conditions. If my reading is correct, then it needs to be highlighted as it I a counterintuitive and potentially very important concept.

I recommend acceptance with minor revisions. The paper is generally well written, the figures, and well-drafted, and the paper is though provoking. The timescales and stick/bounce criteria that the paper presents should be applicable to other systems and help advance our understanding of silicic volcanism. I look forward to seeing it in print.

Benjamin J. Andrews
Smithsonian Global Volcanism Program

Line-by-line comments/edits (approximate line number)
72 – change to “often begin with an explosive phase”
73 – what do you mean by “this type of volcanism”? Be careful how far you carry the generalization in the early part of the manuscript – if you mean for all “silicic eruptions,” then this may be a stretch. If “this type” of volcanism refers to eruptions that also emplace a silicic lava (then say so), and the generalization in this paragraph is probably fine.
74 – “have shown that lava can effuse from the vent while an explosive component of the eruption…”
114 – change to “In addition, report lava veneers from…”
116 – say how many other sites worldwide you report the veneers instead of saying “multiple”
119 – “Better understanding of this process can lead to greater knowledge of conduit processes and improved management of the associated volcanic hazards.”
152 – change “commonly” to “often” or “frequently”
157 – “In addition, we surveyed…”
180 – “comprises”
186-190 – this is a nice interpretation (really – I like it), but it might fit better later in the manuscript in the discussion.
210 – clarify that this is the etching of the microlites from the glass by volcanic gases
215 – “modulate the ash populations produced by fragmentation” or “modify ash populations after fragmentation”
277 – “The largest particles…”
280 – “At those lower velocities…” – I would also suggest that such low flow velocities might have trouble moving the coarser particles through the system at all.
290-304 – what are the fall velocities in air for your various particle sizes? This would provide a useful reference for the speeds and transport processes you are discussing. A very rough estimate of fall velocity suggests that 100 um pumice particles have a fall velocity on the order of 1 m/s, which suggest that you need gas velocity through the fractures that is >1 m/s (probably >>1) if particles larger than 100 um are to be effectively transported. And this is before any of the turbulence processes you have nicely described. In other words, by the time the particles are able to move, they are probably going too fast to stick when they impact.
326 – This is an interesting point. If I understand correctly, the smallest particles record the periods of high energy, whereas the larger particles are deposited during waning flow velocity. This is very interesting, and the opposite of how deposits in, e.g., fluvial settings are interpreted. ! If this is indeed correct, I would emphasize this, as it is very important for the interpretation of fuffisites.
338-350 – This is a generally good paragraph, I would just emphasize that the timescales you get here are minima (as, for example, material could have been removed, and thus the record is incomplete).
385-404 – the basic method you use for estimating the eruption rate, and thus the total mass of ash, is basically sound, with one caveat. The heat from the erupting lava will also contribute to the plume height, thus the plume heights are not solely the result of the erupting hot ash mass, but are also increased by the heat from the lava flow. There isn’t a great way to quantify this, but this contribution (and the resulting reduction in calculated ash mass) should be noted.
406-419 – This is basically reasonable, but you need to explain why if the ~0.4 km3 HRN tephra deposit has been mapped, the comparable volume fine ash deposit from hybrid eruption phase has not. (I would surmise that because it is very fine grained, it dispersed farther and was more easily eroded.)
441 – there is a “?” where I think you need a citation.
460 – I would change the word “performance” to “resolution” or whatever particular parameter(s) you mean.
462 – change “in consistence with” to “consistent with”
463 – indicate the minimum number of particles that were analyzed from the 4 samples (e.g., you measured 2599 particles from 4 samples, with each sample having a minimum of 400 analyzed particles).
473 – “For the purposes of this paper, and considering their limitations, we used the 2D PSDs to inform the range of particle sizes observed in the samples, rather than more detailed analyses of the distributions.”
507 – “Further, we recognize that…”

Supplemental material. Please add detailed captions to each of the figures.

Reviewer #3

(Remarks to the Author)

Understanding the origin of volcanic ash, its flux to the atmosphere, and the duration of ash venting are important for assessing volcanic hazards. This process and these quantities also reveal the degassing and fragmentation dynamics during eruption. The present study documents ash deposits preserved in rhyolite lavas that record sustained ash venting while the lavas were erupting, providing further examples of dynamics recorded (both in lavas and with direct observations) elsewhere.

Overall, the paper is clear, and I do not have much to ask or suggest. To me, the novelty of the present study is the model for the capture and preservation of the ash particles by sticking. Here the velocity is critical. The authors consider some of the constraints required for their turbophoresis explanation to enable collisions with the crack walls. Observationally, at Cordon Caulle it seems that ash venting velocities were typically 5-25 m/s (ref 13). Is there a Stokes number requirement as well to enable particles to make contact with the wall by separating from turbulent eddies? Collisions between particles during ascent is another way to deflect them to crack walls. Looking at the figures in Dufek et al. (Nature Geoscience 2012) it seems the particles could be colliding with each other.

Is it possible to also address the conditions that would lead to erosion? Figure 2g and similar examples reported in ref 21 record erosive events, in addition to fluctuating particles sizes in the layers.

I do have one possibly useful question and suggestion that might help test the sticking model, at least conceptually. Lines 180-181 note that crystal fragments are rare. Is the volume fraction of crystals in the sintered ash lower than the bulk magma? Crystals should bounce, rather than stick, when they impact crack walls, so a test of the sticking model might be a depletion of crystals relative to the bulk.

I also think the manuscript overstates the novelty to the conclusions and the novelty of identifying of ash layers in rhyolite flows. The reference list contains several examples of studies that document something similar and with similar conclusions about the hazard. Thus, words like “emerging” on the 3rd line of the abstract are perhaps a bit strong, as is the concluding paragraph.

Line 480. It seems unusual to single out one author for collecting samples (unless the person who collected the samples is not an author).

AI was not used for any aspect of reading the paper or preparing this review, so all typos and misunderstandings can be attributed to the reviewer.

Version 1:

Reviewer comments:

Reviewer #1

(Remarks to the Author)
This revised version is mostly well presented and clearly points out the possible hazards that may accompany hybrid, silicic eruptions. I recommend publication with minor corrections.

Specific comments:

35-39: The Eyjafjallajökull eruption in 2010 also displayed such hybrid activity over ~2 weeks when a lava propagated under the glacier towards north while 2-4 km high plume carried some tephra. If you want to stick to fully silicic eruptions (SiO_2 very high) you can of course omit this - but it is a recent, observed example.

105: ….and pumiceous pyroclasts and are common…. Remove “and“

132-133: …display moderately-sintering, should this not be moderate sintering?

201: By using the temperature dependence of viscosity μ for the glass particles,….. viscosity of glass particles? Does this mean …of flow consisting of glass particles? Or the viscosity of the material that the “glass“ particles are made off? Not clear.

213: Again, viscosity of glass particles … should this not be viscosity of flow? Please make clear.

237-270: Here the laminar/turbulent transition is discussed and the Reynolds number 2300-2900 stated as defining the transition. However, in the text in lines 237-245, the value stated for the laminar regime being $Re \ll 1$ and for the turbulent regime $Re \gg 1$. This needs to be corrected (easy to do) – if fully laminar for the conditions assumed, Re should be considerably less than 2300 (possibly $Re \ll 2300$) and if fully turbulent $Re \gg 2900$.

237-270 again: The Re ~ 2300-2900 laminar-turbulent transition applies to a pipe. If in a wide conduit (e.g. similar to a "vertical sheet") the Reynolds number for laminar-turbulent transition may be considerably higher (e.g. Incropera and deWitt, 1981). This is probably not of major relevance here, but okay to make clear with perhaps one sentence to make clear that a pipe model is appropriate.

391-392: „we estimate the hybrid phase of HRN to have produced 4.8×10E10 kg of tephra.“ Ok, but this is basically guesswork, poorly supported by any empirical data. Having said that, this may have been the case. What about saying: , the hybrid phase of HRN would have produced 4 x 10E10 kg of tephra. Assuming….. This is more cautious but carries the same message.

Open Access This Peer Review File is licensed under a Creative Commons Attribution 4.0 International License, which permits use, sharing, adaptation, distribution and reproduction in any medium or format, as long as you give appropriate credit to the original author(s) and the source, provide a link to the Creative Commons license, and indicate if changes were made.
In cases where reviewers are anonymous, credit should be given to 'Anonymous Referee' and the source.
The images or other third party material in this Peer Review File are included in the article’s Creative Commons license, unless indicated otherwise in a credit line to the material. If material is not included in the article’s Creative Commons license and your intended use is not permitted by statutory regulation or exceeds the permitted use, you will need to obtain permission directly from the copyright holder.
To view a copy of this license, visit https://creativecommons.org/licenses/by/4.0/

Sustained ash emission during lava effusion is a hidden volcanic hazard of silicic eruptions

Response to reviewers – Mar. 2026

Note to editor

Line numbers have been re-indexed due to migration from LaTeX to Microsoft Word, and may be significantly different to those in the initial submission. Where applicable, we have added (in parenthesis) the equivalent current line numbers (simple markup) next to the original reviewers' comments.

Reviewer #1

Main

This manuscript presents in many ways a new insight into possible style of silicic eruptions where ash emission may accompany/be a major part of what have been considered predominantly effusive eruptions. The Cordon Caulle eruption in Chile in 2011-2012 is used as an example of such events. The possible occurrence of this type of behavior for the ~877 AD Hrafntinnuhraun eruption (the silicic part of the largely basaltic explosive Vatnaöldur eruption) is advocated. The explosive tephra “eruption” is considered to have occurred through fractures in the high viscosity lava directly above the vent. Detailed field descriptions of the evidence for veneers coating fractures in the lava and a conceptual model are presented. Estimates of likely tephra production during these assumed hybrid events (effusive + explosive) are presented and the amount of explosively transported tephra is presented, indicating that its volume, erupted throughout the lifetime of the eruption (of order 150-200 days), may have been similar to the volume of the lava formed in the same event. This idea is most interesting and the results as well. It is advocated that explosive activity/tephra production (apparently predominantly fine ash) occurring during the effusive phases of these silicic eruptions is a hazard that has not been properly identified before.

Although this is highly interesting, it has to be said that there is little empirical evidence for such a significant explosive phase occurring at the same time as the silicic lava eruption at the location studied (Hrafntinnuhraun). In known explosive eruptions of moderate/low intensity, the dispersive power is much less than in more powerful eruptions. As a consequence, tephra tends to pile up in the vicinity of the vents. Also, if the volumetric flow rate of tephra was of the same order of magnitude as that of the lava (~half a cubic kilometer), a sizeable tephra layer would be found in the region, as well as prominent craters. If deposited mostly locally (due to low intensity) the latter would probably be dominant. Moreover, if similar to 0.5 km3 of tephra was erupted over 180 days (density 600 kg/m3) the average tephra MER would have been similar to 200,000 kg/s. This would have been almost twice the average MER in the 39-day long Eyjafjallajökull eruption in 2010 (3.8 x 10pow11 kg ~ 120,000 kg/s). The 2010 Eyjafjallajökull tephra layer is well preserved in soil in a large region extending tens of kilometers from the volcano. Preservation potential in the 9th century AD would have been considerably better than today, when birch/tree cover was dominant on the lowlands.

For the 877 eruption a silicic tephra layer is found underneath the much larger basaltic layer formed at the long fissure to the north of Torfajökull (see maps and descriptions in Larsen, 1984). This shows that at least the main explosive silicic phase was not long-lived and occurred at the beginning of the main explosive phase. Hence, the authors should be more careful in their assertions on the relevance of co-effusive explosive activity for this eruption. The empirical data seem to indicate that any co-lava effusion explosive activity was orders of magnitude smaller – airphotos (e.g. www.map.is) and field observations suggest that there is relatively little tephra covering Hrafntinnuhraun. In contrast, on air photos of Cordon Caulle (e.g. google-earth), tephra is quite prominent, apparently covering parts of the lava.

The above is to an extent discussed in line 394-404. However, if the volume produced was similar to that suggested, parts of it should be visible in some of the hundreds of tephra sections studied over several decades in Iceland. If this occurred over extended period, the tephra should be on top of the 877 basaltic tephra formed in the main phase of this eruption. But it is not observed. I therefore suggest that the manuscript should be updated where the nuances/empirical evidence discussed above are fully taken into account.

We are thankful for the astute and important comments regarding the "missing" lava-vented ash that may be expected to be found both proximally and distally.

The reviewer uses calculations to compare with the EYJ2010 tephra - in terms of duration, volume, and flux, suggesting that the Hrafntinnuhraun lava-venting flux would be similar to the mean EYJ2010 flux of 1.12x10^5 kgs-1. We have repeated this calculation and note that theirs is larger by approximately an order of magnitude:

$$0.5\ \text{km}^3 * 600\ \text{kg m}^{-3} = 3\times10^{11}\ \text{kg};$$

$$3\times10^{11}\text{kg}/(180\ \text{days} * 24\ \text{hours/day} * 3600\ \text{seconds/hour}) = 1.9\text{E}4\ \text{kg s}^{-1}$$

and that the sustained ash flux we are anticipating is only ~10 % of the EYJ2010 value, which is similar to the forward calculation used in the manuscript.

The reviewer mentions that tephra is quite prominent on the 2011-12 Cordón Caulle lava flow field, apparently covering parts of the lava. We agree, but there is an important difference – this is not tephra that directly fell onto the lava during hybrid activity, but instead fragments of the now-breached tephra cone that collapsed onto the lava and were rafted down the flow channels.

We agree it is essential to understand why there is no documented evidence to date for any signature of prolonged silicic tephra deposition from HRN877. We have further reflected on this thanks to the reviewer challenge, and have added a substantial new section to the discussion.

When considering this absence, we have first taken the cautious approach and adopted the lower bound of tephra emissions and thus total volume in our forward calculations. This is by assuming that HRN's hybrid phase had an ash venting MER that was the same as the time-averaged, lower-bound scenario MER of Cordón Caulle's ash venting (3.3×10^3 kg s-1). This results in a lower best-estimate total output, ~0.08 km^3, which is ~1/5 of the volume of HRN's Plinian phase output. Importantly, the modelled mass flux of this tephra, at 3.3×10^3 kg s-1, was only ~3% of the equivalent EYJ2010 flux, which *was* distally preserved. Our discussion on preservation then considers this lower volume and significantly lower mass flux, which we argue would have yielded a far lower preservation potential for HRN877, compared to EYJ2010 (discussed in the manuscript lines 396-404, pasted below):

> While the distal tephra stratigraphy of HRN is fairly well studied in Iceland[24], there is no confirmed evidence of the hybrid-phase HRN ash in distal deposits. We suggest that a combination of factors likely contribute to the absence of this tephra, including:

> (1) its gradual deposition at low MER over an extended time period, making syn-depositional remobilization more influential than for rapidly-emplaced tephra; (2) the ash plume will have had a narrow axis of deposition that will have migrated through time with changing wind directions, increasing dispersion and thus reducing preservation potential, and (3) given the long duration and location of eruption, the deposition is likely to have been affected by snowfall and subsequent thawing, which will further reduce its tephra preservation potential. Due to large uncertainties with preservation potential from past eruptions, it may also be the case that the deposit from this phase has been overlooked[60]. We suggest that for comparable past eruptions, their hybrid phases would have similarly produced a sizeable quantity of tephra that is not preserved and/or accurately assessable, hence absent from the record as a unique and identifiable discrete layer.

Finally, our reduced estimate of total hybrid-phase tephra output does not substantially diminish most hazards that we had argued to be associated with ash venting. As was at Cordon Caulle, the hybrid-phase plume heights were greatly variable, with the higher plumes significantly more hazardous, relating to the Jan 2012 closure of Bariloche airport, Argentina. Note that the Cordon Caulle hybrid-phase ash was also poorly preserved.

We hope these updates address your comment adequately.

Minor comments

79-~100 (50-~71): Consider the beginning phases of the Hekla eruptions - they are typically mixed eruptions with explosive phase being dominant to begin with, while effusive activity takes over after the first few hours – however, some tephra is at times deposited for weeks (e.g. 1947).

Thank you for the suggestion. While we agree it is a similar hybrid eruption, Hekla 1947 is intermediate in composition, so we had omitted it for clarity.

115: According to the geological map of Iceland, Hrafntinnusker is considered ~9000 years old. See: https://arcgisserver.isor.is/?lon=-19.29484&lat=63.96733&zoom=12&layers%5B%5D=satellite&layers%5B%5D=geologyDetailed

Corrected for Hrafntinnusker (line 108); the instance originally at line 115 was removed for conciseness.

116: This eruption has been dated to 1477 AD – see e.g. www.icelandicvolcanos.is - look upp Barðarbunga – volcano information – 11. Lagest known eruption. This has not been properly documented in peer-reviewed papers but is based on a written letter from Eyjafjörður in North Iceland in 1477, describing the tephra fallout. Recorded in e.g. Náttúruvá á Íslandi, Eldgos og jarðskjálftar, p. 257, where a citation to the source is given.

Corrected for Laugahraun and Námshraun (line 109), now citing the entry in the Catalogue of Icelandic Volcanoes; the instance originally at line 116 was removed for conciseness.

258 (199): 1.341 x 10 4 Pa - four significant digits? Given the uncertainties, 1.3 is more appropriate than 1.341.

Implemented. (3.341 -> 3.3)

268 (208); 7.09 more appropriate to use 7.1 (see above – significant digits)

Implemented.

415-419: 0.48 km3 - if this value is correct, where is the tephra? Hundreds of tephra layers, most smaller than this volume are preserved in the soils from the Holocene time in Iceland. This value is larger than that for the Eyjafjallajökull eruption of 2010…..

(Addressed in our response to the Main comment above.)

440-444: Cite sources for Eyjafjallajökull tephra volume. The number given comes from Gudmundsson et al. 2012. Scientific Reports https://www.nature.com/articles/srep00572

Corrected, was caused by a LaTeX error.

Figure 1: Not a major issue, but the paper would be stronger if a proper map showing the location and context of the eruption site/Hrafntinnuhraun would be shown. The current map is not very informative/showing the context + the link of the eruption with the main eruption site of Vatnaöldur.

Thank you for the suggestion; however, as our manuscript does not mention the basaltic component of the Vatnaöldur eruption, we've opted not to add it to our map for clarity.

657: The reference is incomplete – should be:

Larsen, G. 1984. Recent volcanic history of the Veidivötn fissure swarm, south Iceland – an approach to volcanic risk assessment. Journal of Volcanology and Geothermal Research, 22, 33-58.

Implemented.

Reviewer #2

Reviewer: Benjamin J. Andrews, Smithsonian Global Volcanism Program

The authors describe fine grained veneer and tuffisite deposits on surfaces and fractures from the Hrafntinnuhraun flow in Iceland. They interpret those deposits are recording transport of fine-grained ash through the fractures with finer grained material sintering on the margins during periods of high velocity gas flow, and particle size increasing as the gas flow wanes. They estimate that individual fractures likely remain open for many hours to days, suggesting that continuous emission of ash can occur from silicic lava flows. Through analogy with the Cordon Caulle eruption, the authors estimate that about 0.48 km3 of fine ash was erupted during the ~6 month eruption timescale of the lava. Finally, they point out that this type of eruption could produce sustained and substantial hazards to local and regional communities, but that this hazard is largely unrecognized.

There are several big ideas that the paper raises. First, the authors bring in the concept of turbophoresis – whereby particles migrate to regions of lower turbulence intensity – in the interpretation of the deposits. Second, the authors explain how and why particles should stick and sinter within the fractures versus why they escape – importantly, they quantify this process to explain the granulometry of the deposits in the fractures/veneers and why observed "extra vent" deposits at Cordon Caulle are ~100um. Together, the turbophoresis and stick-bounce criteria that the authors develop have interesting implications for thinking about ash transport through and deposition within fractures, and for volcanic conduit processes in general.

There are two or three areas in which the paper can be strengthened. First, the authors make a big deal out of turbophoresis, but this concept or process could be explored in a little more detail. Specifically, what types of turbulence intensities (and cross-fracture gradients in turbulence) might we expect to ee in these fractures? The turbulent velocity distributions, and the timescales over which those fluctuations occur, are probably more important for determining deposition of the particles than the mean flow velocities. Second, there is a minimum flow velocity required to transport particles of a given size and density, and this velocity is almost certainly greater than the maximum "sticking" velocity of the larger particles. As a result, once particles become sufficiently large, they basically cannot stick onto the fracture margins. This mobilization velocity is basically the particle settling or fall velocity, and could be plotted on Figure 5 as a line that increases with size (rather than decreasing like the sticking velocity) to show a maximum particle size. Finally, the paper suggests that during intervals of high gas velocity, only small particles are deposited, whereas larger particles get deposited as velocity wanes. This is a big deal as it is more or less the opposite of fluvial processes (large particles indicate high fluid velocities, and fine muds indicate low-velocity or slack conditions. If my reading is correct, then it needs to be highlighted as it I a counterintuitive and potentially very important concept.

I recommend acceptance with minor revisions. The paper is generally well written, the figures, and well-drafted, and the paper is though provoking. The timescales and stick/bounce criteria that the paper presents should be applicable to other systems and help advance our understanding of silicic volcanism. I look forward to seeing it in print. Benjamin J. Andrews

Smithsonian Global Volcanism Program

We thank the reviewer for their comments, compliments, and recommendation for publication in Nature Communications. We address their suggestions below through their line-by-line review.

Line-by-line comments/edits (approximate line number)

72 (35)– change to “often begin with an explosive phase”

Implemented.

73 – what do you mean by “this type of volcanism”? Be careful how far you carry the generalization in the early part of the manuscript – if you mean for all “silicic eruptions,” then this may be a stretch. If “this type” of volcanism refers to eruptions that also emplace a silicic lava (then say so), and the generalization in this paragraph is probably fine.

“this type of” removed; the original sentence reworked for clarity.

74 (37) – “have shown that lava can effuse from the vent while an explosive component of the eruption…”

Implemented.

114 (73)– change to “In addition, report lava veneers from…”

Reworked.

116 – say how many other sites worldwide you report the veneers instead of saying “multiple”

Reworked, now lists all sites reported.

119 (77) – “Better understanding of this process can lead to greater knowledge of conduit processes and improved management of the associated volcanic hazards.”

Implemented.

152 (102) – change “commonly” to “often” or “frequently”

Implemented.

157 (108) – “In addition, we surveyed…”

Implemented.

180 (129) – “comprises”

Implemented.

186-190 – this is a nice interpretation (really – I like it), but it might fit better later in the manuscript in the discussion.

Thanks! Implemented. – now placed in Discussion, line 298-303.

210 (153-154)– clarify that this is the etching of the microlites from the glass by volcanic gases

Implemented.

215 (158) – “modulate the ash populations produced by fragmentation” or “modify ash populations after fragmentation”

Implemented.

277 (219)– “The largest particles…”

Implemented.

280 – “At those lower velocities…” – I would also suggest that such low flow velocities might have trouble moving the coarser particles through the system at all.

(Please see our reply to the following comment.)

290-304 – what are the fall velocities in air for your various particle sizes? This would provide a useful reference for the speeds and transport processes you are discussing. A very rough estimate of fall velocity suggests that 100 um pumice particles have a fall velocity on the order of 1 m/s, which suggest that you need gas velocity through the fractures that is >1 m/s (probably >>1) if particles larger than 100 um are to be effectively transported. And this is before any of the turbulence processes you have nicely described. In other words, by the time the particles are able to move, they are probably going too fast to stick when they impact.

Thank you for your comment. We initially removed the settling velocity comparisons as they were consistently lower than the turbulence velocities, for the smallest particles.

Nevertheless, we agree that this is a useful comparison – as such, we have now added the settling velocities (both Re<<1 and Re>>1) to the discussion (at lines 258-277). We note that we’re calculating these velocities for vesicular particles, within conduit-like conditions, as with the rest of our modelling approach. With these parameters, a 100 µm diameter particle has settling velocities of 0.41 ms-1 (Re<<1) and 0.35 ms-1 (Re>>1).

We hope these updates address your comment adequately.

326 – This is an interesting point. If I understand correctly, the smallest particles record the periods of high energy, whereas the larger particles are deposited during waning flow velocity. This is very interesting, and the opposite of how deposits in, e.g., fluvial settings are interpreted. ! If this is indeed correct, I would emphasize this, as it is very important for the interpretation of fuffisites.

Thank you. The seeming discrepancy arises because the dominant sorting processes differ fundamentally between open-channel flows and confined particle-laden jets. In fluvial systems, grains are transported in a gravity-driven shear flow with a velocity gradient from bed to surface. Coarse grains require higher boundary shear stress to be mobilized: a dependence that is well established (e.g. Buffington and Montgomery 1997; van Rijn et al., 1984a,b). As flow velocity decreases, the available shear stress similarly decreases and reduces the transport capacity of the flow (carrier phase). Coarser particles, which have higher settling u and are less easily suspended, are therefor deposited first under waning flow conditions (e.g. Ferguson and Church, 2004). Fine(r) particles remain in suspension until flow energy drops even more, producing the characteristic coarse-to-fine depositional sequence.

In a nominally vertical tuffisite-forming conduit/fracture, the controlling processes should be lateral particle migration (i.e. sideways transport) and capture of ash, not just vertical settling. Turbophoresis serves to enhance the former, while sintering accounts for the latter. Concentrated (sub)micron particles can sinter rapidly, as our ash-capture model shows, whereas at higher velocities, larger particles are more likely to rebound and be re-entrained into the flow. So while rivers and similar sedimentological deposition systems sort by settling under gravity, we model conduits as “particle filters” driven by turbulence-enhanced wall capture such that fine particles are preferentially “deposited” during the energetic phases of transport/flow.

However, as shown in Figures 2b, 2h, we do occasionally see more intricate depositional patterns in these veneers and tuffisites (e.g. dunes or ripples). This may well point to an additional complexity in the tortuous fracture pathways: at some stages of the eruption, the depositional regime may have been closer to classic settling dynamics. This would superimpose fluvial-style sorting onto the turbulence-governed depositional framework.

To allow for this to be appropriately explored in the manuscript, we have now elaborated further in the manuscript, at lines 284-292.

References for the above:

Buffington, J.M. and Montgomery, D.R., 1997. A systematic analysis of eight decades of incipient motion studies, with special reference to gravel‐bedded rivers. Water resources research, 33(8), pp.1993-2029.

Ferguson, R.I. and Church, M., 2004. A simple universal equation for grain settling velocity. Journal of sedimentary Research, 74(6), pp.933-937.van Rijn, L.C., 1984a. Sediment transport, part I: bed load transport. *Journal of hydraulic engineering*, *110*(10), pp.1431-1456.

van Rijn, L.C., 1984b. Sediment transport, part II: suspended load transport. *Journal of hydraulic engineering*, *110*(11), pp.1613-1641.

338-350 (305-320) – This is a generally good paragraph, I would just emphasize that the timescales you get here are minima (as, for example, material could have been removed, and thus the record is incomplete).

Implemented.

385-404 (352-368)– the basic method you use for estimating the eruption rate, and thus the total mass of ash, is basically sound, with one caveat. The heat from the erupting lava will also contribute to the plume height, thus the plume heights are not solely the result of the erupting hot ash mass, but are also increased by the heat from the lava flow. There isn’t a great way to quantify this, but this contribution (and the resulting reduction in calculated ash mass) should be noted.

We haven’t found a source suggesting that this effect is relevant for moderate-scale silicic eruptions. Moreover, the potential thermal effect from the lava was not recognized at Cordon Caulle by Bonadonna et al. 2015 (ref. 59 of the main manuscript).

406-419 (370-380) – This is basically reasonable, but you need to explain why if the ~0.4 km3 HRN tephra deposit has been mapped, the comparable volume fine ash deposit from hybrid eruption phase has not. (I would surmise that because it is very fine grained, it dispersed farther and was more easily eroded.)

We’ve updated our estimates using a more conservative approach, in order to take into account the empirical observation of the missing hybrid-phase tephra from HRN. Our revised best estimate is 0.08 km^3. Additionally, we have made a more detailed discussion of the factors that likely contributed to a low preservation potential for this tephra (line 382-395).

441 (418) – there is a “?” where I think you need a citation.

Corrected, was caused by a LaTeX error.

460 (432)– I would change the word “performance” to “resolution” or whatever particular parameter(s) you mean.

Implemented.

462 (435) – change “in consistence with” to “consistent with”

Implemented.

463 (443)– indicate the minimum number of particles that were analyzed from the 4 samples (e.g., you measured 2599 particles from 4 samples, with each sample having a minimum of 400 analyzed particles).

Implemented.

Corrected: 4-> 3 samples, 2599->2639 particles. The errors were in text only – the original manuscript made use of the corrected numbers of samples and particles.

473 (447) – “For the purposes of this paper, and considering their limitations, we used the 2D PSDs to inform the range of particle sizes observed in the samples, rather than more detailed analyses of the distributions.”

Implemented.

507 (474) – “Further, we recognize that…”

Sentence reworked.

Supplemental material. Please add detailed captions to each of the figures.

Implemented.

Reviewer #3

Understanding the origin of volcanic ash, its flux to the atmosphere, and the duration of ash venting are important for assessing volcanic hazards. This process and these quantities also reveal the degassing and fragmentation dynamics during eruption. The present study documents ash deposits preserved in rhyolite lavas that record sustained ash venting while the lavas were erupting, providing further examples of dynamics recorded (both in lavas and with direct observations) elsewhere.

Overall, the paper is clear, and I do not have much to ask or suggest. To me, the novelty of the present study is the model for the capture and preservation of the ash particles by sticking. Here the velocity is critical. The authors consider some of the constraints required for their turbophoresis explanation to enable collisions with the crack walls. Observationally, at Cordon Caulle it seems that ash venting velocities were typically 5-25 m/s (ref 13). Is there a Stokes number requirement as well to enable particles to make contact with the wall by separating from turbulent eddies? Collisions between particles during ascent is another way to deflect them to crack walls. Looking at the figures in Dufek et al. (Nature Geoscience 2012) it seems the particles could be colliding with each other.

We agree that particle inertia (i.e. as quantified by the Stokes number) is an important constraint on wall collision “efficiency”. While turbophoresis can drive fine ash towards low-turbulence/laminar regions at the conduit/fracture margins, their effective capture relies on full or partial decoupling from turbulent flow structures (eddies). For the Cordón Caulle eruption, Farquharson et al. (2022; ref 9 in main manuscript) demonstrated that ash particles in the micron/submicron range occupied an optimal parameter regime where both turbophoretic lateral migration (towards the wall) and inertial impaction due to decoupling from the carrier phase were operative and effective. We deem it highly probable that particle disruption (*sensu* Dufek et al. 2012; ref 32 in main manuscript) would further enhance wall interactions (and the fraction of fines), both at Cordón Caulle and during the Hrafntinnuhraun eruption.

Farquharson et al. also demonstrated that gas velocities in the range ~10–100 m s-1 were consistent with turbophoresis-driven fine-ash capture based on the inferred Stokes and Weber–Ohnesorge regimes. In carrier phase velocity (u) versus ash radius (R) space, the calculated Stokes number reaches unity only within a bounded region, defined by maximum and minimum limits corresponding to the outer and Kolmogorov scales, respectively. A Stokes number of 1 cannot occur out with this range; within it, particles can be dynamically decoupled from the carrier phase and cluster in the flow: not so large that they are on ballistic trajectories, and not so small that they follow streamlines (i.e. complete coupling). This is the range where turbophoresis should be most effective.

Is it possible to also address the conditions that would lead to erosion? Figure 2g and similar examples reported in ref 21 record erosive events, in addition to fluctuating particles sizes in the layers.

We could not find a suitable, experimentally-backed model for this behavior, so did not include in our modelling.

I do have one possibly useful question and suggestion that might help test the sticking model, at least conceptually. Lines 180-181 note that crystal fragments are rare. Is the volume fraction of crystals in the sintered ash lower than the bulk magma? Crystals should bounce, rather than stick, when they impact crack walls, so a test of the sticking model might be a depletion of crystals relative to the bulk.

Thank you for the suggestion, but HRN lava is also crystal poor in general, and any comparisons would need to account for crystallization during the more prolonged cooling of the lava. Nevertheless, this could perhaps be something to be investigated at a different volcanic system.

I also think the manuscript overstates the novelty to the conclusions and the novelty of identifying of ash layers in rhyolite flows. The reference list contains several examples of studies that document something similar and with similar conclusions about the hazard. Thus, words like “emerging” on the 3rd line of the abstract are perhaps a bit strong, as is the concluding paragraph.

Re-phrased, where applicable.

Line 480. It seems unusual to single out one author for collecting samples (unless the person who collected the samples is not an author).

Removed.

AI was not used for any aspect of reading the paper or preparing this review, so all typos and misunderstandings can be attributed to the reviewer.

Sustained ash emission during lava effusion is a hidden volcanic hazard of silicic eruptions

Response to reviewers – Jul. 2026

We are thankful for the perceptive and helpful reviewer comments and have made appropriate modifications as summarised below.

REVIEWERS' COMMENTS

Reviewer #1 (Remarks to the Author):

This revised version is mostly well presented and clearly points out the possible hazards that may accompany hybrid, silicic eruptions. I recommend publication with minor corrections.

Specific comments:

35-39: The Eyjafjallajökull eruption in 2010 also displayed such hybrid activity over ~2 weeks when a lava propagated under the glacier towards north while 2-4 km high plume carried some tephra. If you want to stick to fully silicic eruptions (SiO_2 very high) you can of course omit this - but it is a recent, observed example.

Thank you for this suggestion. We have added a reference to the 2010 Eyjafjallajökull eruption as:

"…lava can effuse from the vent while an explosive component of the eruption remains active, in the form of low- to moderate intensity ash venting. Similar behavior can also manifest in lower silicic magma including the 2010 eruption of Eyjafjallajökull, Iceland, during which weak but sustained 2–4 km high ash plumes accompanied lava effusion[10]."

105: ….and pumiceous pyroclasts and are common…. Remove "and"

Implemented.

132-133: …display moderately-sintering, should this not be moderate sintering?

Implemented.

201: By using the temperature dependence of viscosity μ for the glass particles,….. viscosity of glass particles? Does this mean …of flow consisting of glass particles? Or the viscosity of the material that the "glass" particles are made off? Not clear.

We refer to the latter – the viscosity of the glass itself. The text has been modified to clarify:

"…using the temperature dependence of viscosity μ for the glass particles (i.e. the viscosity of the glass itself, not of the flow containing the particles), we can…"

213: Again, viscosity of glass particles … should this not be viscosity of flow? Please make clear.

We believe this comment is resolved by addressing the previous comment.

237-270: Here the laminar/turbulent transition is discussed and the Reynolds number 2300-2900 stated as defining the transition. However, in the text in lines 237-245, the value stated for the laminar regime being Re << 1 and for the turbulent regime Re>>1. This needs to be corrected (easy to do) – if fully laminar for the conditions assumed, Re should be considerably less than 2300 (possibly Re << 2300) and if fully turbulent Re >> 2900.

The reviewer has raised an interesting point. We state that fully developed turbulence onsets around Re=2900, but then shortly after (our line 274) we state that turbulence occurs at Re>>1. Both are true since Re=2900 is Re>>1, but the reviewer is also correct that the latter statement (Re>>1) could be more precise. As such, we now replace all instances of "Re>>1" and "Re<<1" with "Re>2900" and "Re<2300", respectively, in both the main text and figure captions.

237-270 again: The Re ~ 2300-2900 laminar-turbulent transition applies to a pipe. If in a wide conduit (e.g. similar to a "vertical sheet") the Reynolds number for laminar-turbulent transition may be considerably higher (e.g. Incropera and deWitt, 1981). This is probably not of major relevance here, but okay to make clear with perhaps one sentence to make clear that a pipe model is appropriate.

We have now clarified this in the text as:

"Fully developed turbulence is expected at Re>2900, while laminar flow is expected at Re<2300 [39]. We note that these thresholds are specific to flow in a pipe, which we use as a reference case for ascent through the shallow surface, but we acknowledge that the exact threshold in terms of Re for fully developed turbulence could be different (e.g. depending on surface roughness or geometry differences such as flow up a crack rather than a pipe). The order of magnitude of these transitions tends to be similar such that turbulence occurs at Re> O(10^3)."

391-392: „we estimate the hybrid phase of HRN to have produced 4.8×10E10 kg of tephra." Ok, but this is basically guesswork, poorly supported by any empirical data. Having said that, this may have been the case. What about saying: , the hybrid phase of HRN would have produced 4 x 10E10 kg of tephra. Assuming….. This is more cautious but carries the same message.

We have rephrased the sentence as suggested.
